# ITGB1-DT/ARNTL2 axis may be a novel biomarker in lung adenocarcinoma: a bioinformatics analysis and experimental validation

**DOI:** 10.1186/s12935-021-02380-2

**Published:** 2021-12-14

**Authors:** Bai-Quan Qiu, Xia-Hui Lin, Song-Qing Lai, Feng Lu, Kun Lin, Xiang Long, Shu-Qiang Zhu, Hua-Xi Zou, Jian-Jun Xu, Ji-Chun Liu, Yong-Bing Wu

**Affiliations:** 1 Department of Cardiothoracic Surgery, The Second Affiliated Hospital of Nanchang University, Nanchang, Jiangxi China; 2 Key Laboratory of Carcinogenesis and Cancer Invasion, Ministry of Education, Shanghai, China; 3 Institute of Cardiovascular Disease, Jiangxi Academy of Clinical Medical Sciences, The First Affiliated Hospital of Nanchang University, Nanchang, Jiangxi China

**Keywords:** LUAD, ceRNA, ITGB1-DT/ARNTL2 axis, Prognosis, Public database, Proliferation, Invasion

## Abstract

**Background:**

Lung cancer is one of the most lethal malignant tumors that endangers human health. Lung adenocarcinoma (LUAD) has increased dramatically in recent decades, accounting for nearly 40% of all lung cancer cases. Increasing evidence points to the importance of the competitive endogenous RNA (ceRNA) intrinsic mechanism in various human cancers. However, behavioral characteristics of the ceRNA network in lung adenocarcinoma need further study.

**Methods:**

Groups based on SLC2A1 expression were used in this study to identify associated ceRNA networks and potential prognostic markers in lung adenocarcinoma. The Cancer Genome Atlas (TCGA) database was used to obtain the patients' lncRNA, miRNA, and mRNA expression profiles, as well as clinical data. Informatics techniques were used to investigate the effect of hub genes on prognosis. The Cox regression analyses were performed to evaluate the prognostic effect of hub genes. The methylation, GSEA, and immune infiltration analyses were utilized to explore the potential mechanisms of the hub gene. The CCK-8, transwell, and colony formation assays were performed to detect the proliferation and invasion of lung cancer cells.

**Results:**

We eventually identified the ITGB1-DT/ARNTL2 axis as an independent fact may promote lung adenocarcinoma progression. Furthermore, methylation analysis revealed that hypo-methylation may cause the dysregulated ITGB1-DT/ARNTL2 axis, and immune infiltration analysis revealed that the ITGB1-DT/ARNTL2 axis may affect the immune microenvironment and the progression of lung adenocarcinoma. The CCK-8, transwell, and colonu formation assays suggested that ITGB1-DT/ARNTL2 promotes the progression of lung adenocarcinoma. And hsa-miR-30b-3p reversed the ITGB1/ARNTL2-mediated oncogenic processes.

**Conclusion:**

Our study identified the ITGB1-DT/ARNTL2 axis as a novel prognostic biomarker affects the prognosis of lung adenocarcinoma.

**Supplementary Information:**

The online version contains supplementary material available at 10.1186/s12935-021-02380-2.

## Introduction

Lung cancer is the second most commonly diagnosed cancer and the leading cause of cancer death worldwide [1], and lung adenocarcinoma (LUAD) is the most common subtype of lung cancer, accounting for approximately 40% of all lung cancer cases [2, 3]. However, difficulties in early diagnosis and multiple complications result in a mortality rate of up to 80% [4]. Despite recent advances in immunotherapy, LUAD still responds inconsistently to immunotherapy [5]. There are few strategies for the prevention or early treatment of LUAD due to the limited number of specific targets found in the disease [6].

Currently, The Cancer Genome Atlas (TCGA) is the most comprehensive cancer molecular and clinical database [7, 8]. Many candidate biomarkers for multiple cancers have been discovered with the help of the TCGA database [9–11]. So far, more and more non-invasive, repeatable, and accurate tools for early patient screening and diagnosis have been developed with the support of high-throughput RNA sequencing (RNA-seq) technology.

Non-coding RNAs (ncRNAs) were previously assumed to be transcription noise, however, a growing body of research suggests that they may play an important role in the transition from normal to disease [12–14]. Long non-coding RNAs (lncRNAs) are a type of non-coding RNAs that is longer than 200nucleotides (nt). It can cause mRNA degradation or translation repression by sponging miRNAs (a type of small single-strand ncRNA with 19–26 nt) [15–17]. According to recent research, lncRNAs have both direct and indirect regulatory effects in the biological process of cancer [18–20]. Also, it has been reported that lncRNAs are related to the recurrence, metastasis, and prognosis of LUAD [21–24].

Solute carrier family 2 member 1 (SLC2A1, also known as GLUT, GLUT-1, and so on) is a common oncogene in multiple cancers [25–28], including LUAD [29]. Meanwhile, some evidence suggests that increased SLC2A1 expression is involved in glycolysis [25], cell proliferation [30], cancer growth, and metastasis [28], and is also related to the prognosis of LUAD [31, 32].

**Fig. 1:**
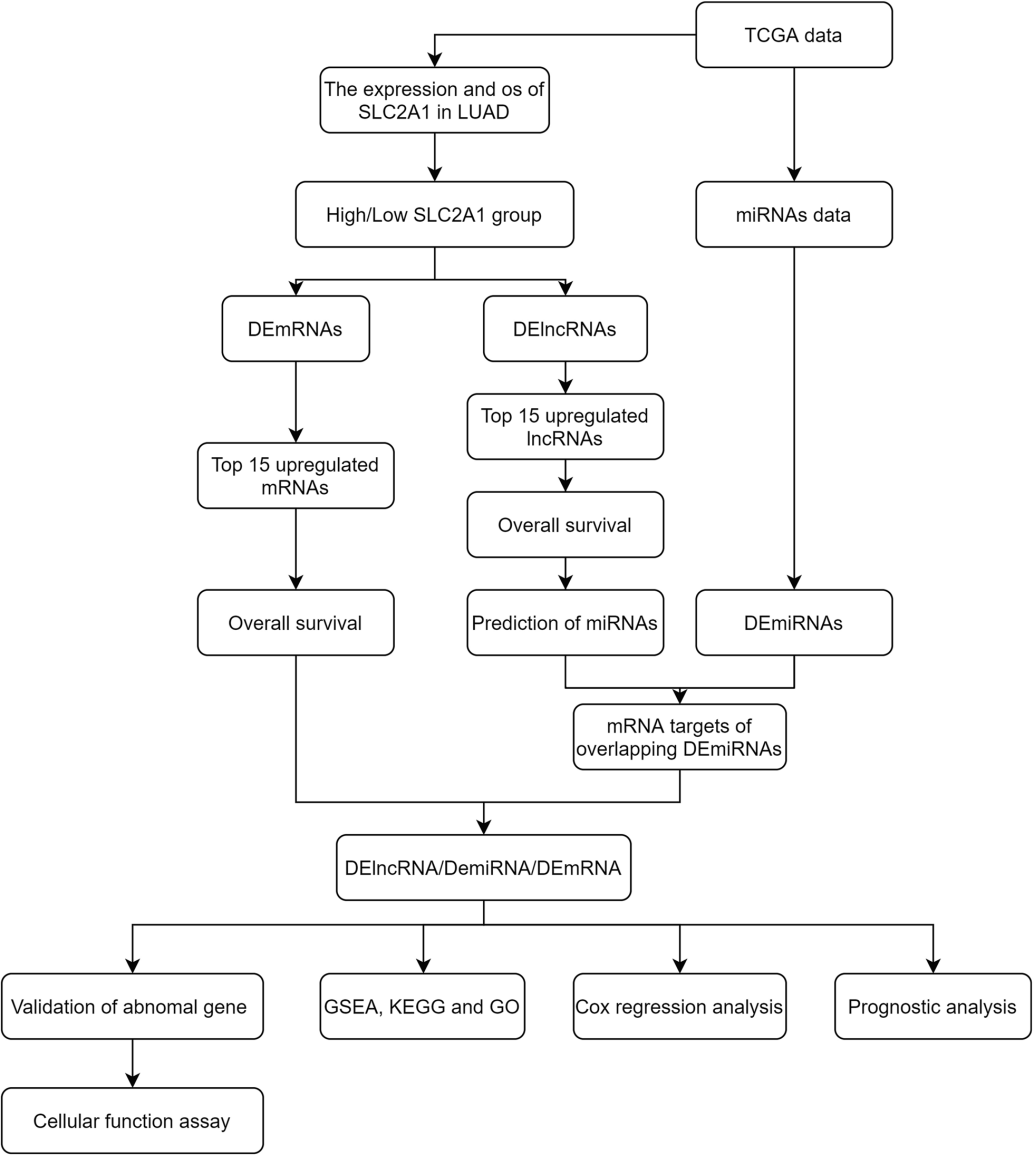
The flowchart of whole study

In our study, the flow chart is depicted in Fig. 1. First, the differentially expressed lncRNAs (DElnRNAs) and mRNAs (DEmRNAs) were screened in two groups with high SLC2A1 (first 25% LUAD samples, n = 133) and low SLC2A1 (last 25% LUAD samples, n = 133) expression in LUAD. Differentially expressed miRNAs (DEmiRNAs) were identified in tumor and peri-tumor samples. Then, we constructed a ceRNA network and used survival analysis to identify the key genes. The findings suggested that ITGB1-DT/ARNTL2 axis may play a critical role in LUAD. Cox regression analysis was performed to evaluate the prognostic values of ARNTL2, methylation analysis, and immune infiltration analysis were used to determine the potential function of ARNTL2 in LUAD. Using Gene set enrichment analysis (GSEA) to further explore the mechanism of ARNTL2. Nomogram was used for prognostic judgment.

## Materials and methods

### Data source and preparation

Figure 1 depicts the workflow chart of the whole study. The primitive counts data, mature miRNAs data, and clinical information were downloaded from the TCGA (https://portal.gdc.cancer.gov/) database. The DElnRNAs and DEmRNAs were screened with the “DESeq2” R package [33], and the DEmiRNAs were screened with the “Limma” package [34]. The lncRNAs and mRNAs RNA-seq data were normalized as transcripts per million reads (TPM), while the miRNA RNA-seq data were normalized as reads per million mapped reads (RPM). The cilinical data were supplemented in the original data.

To validate our findings, we downloaded the expression matrix from the Gene Expression Omnibus (GEO: https://www.ncbi.nlm.nih.gov/geo/) database: GSE43458 (including 80 LUAD tissues and 30 normal lung tissues; platform: GPL6244). The protein expression of SLC2A1 was obtained from The Human Protein Atlas (HPA) database (https://www.proteinatlas.org/).

### Screening of differentially expressed genes

The differential expression analysis of DElncRNAs and DEmRNAs was performed in SLC2A1^high^ and SLC2A1^low^ LUAD samples. The screening threshold of DElncRNAs and DEmRNAs is |logFC|> 1 and adjusted P-value < 0.05. The differential expression analysis of DEmiRNAs was performed in tumor and peritumor LUAD tissues, and the screening threshold is |logFC|> 0.5 and adjusted P-value < 0.05. The volcano plots and single-gene co-expression heatmap were visualized by the “ggplot2” R package, and the heatmap clustering was drawn by the “ComplexHeatmap” R package [35].

### Construction of the ceRNA network in LUAD

It has been reported that competitive binding of lncRNA to miRNA in the cytoplasm could indirectly regulate mRNA expression, and the following are the ways to construct a ceRNA network: (1) The potential target miRNAs of DElncRNAs were predicted using LncBase Predicted v2.0 database (http://carolina.imis.athena-innovation.gr/diana_tools/web/index.php?r=lncbasev2%2Findex-predicted). (2) The potential mRNAs of DEmiRNAs were predicted using miRWalk 2.0 (http://mirwalk.umm.uni-heidelberg.de/). (3) The Venn diagrams were drawn to show the overlapping gene between the predicted targets and DEGs (https://bioinfogp.cnb.csic.es/tools/venny/). (4) The lncRNA-miRNA-mRNA regulatory network was constructed for further analysis.

The lncRNA sequences were obtained from the LNCipedia database (https://lncipedia.org/) and the cellular localization of lncRNAs were identified in the lncLocator database (http://www.csbio.sjtu.edu.cn/bioinf/lncLocator/).

### Functional enrichment analysis

The GSEA analysis, GO, and KEGG were conducted with TCGA-LUAD dataset by the “Clusterprofiler” R package [36]. The LUAD samples were divided into 2 groups based on the median expression of hub genes, and the C2.cp.v7.2.symbols.gmt [Curated] downloaded from MSigDB Collections (http://software.broadinstitute.org/gsea/msigdb/index.jsp) was chosen as the reference gene sets for GSEA analysis [37]. False discovery rate (FDR) < 0.25 and adjusted P-value < 0.05 were considered significantly.

### Survival and prognosis analysis

The TCGA clinical dataset was used to analyze the LUAD patients’ survival outcomes. To examine the link between DElncRNAs, DEmiRNAs, and DEmRNAs in the ceRNA regulatory network and the overall survival of LUAD patients, we used GraphPad Prism 8 software to depict the scatter plots for analysis and used “survminer” and “survival” R packages to perform the Kaplan–Meier analysis and Log-Rank test. The association between candidate genes in the ceRNA network and overall survival was investigated using Univariate Cox Regression analysis and Multivariate Cox Regression analysis. A log-rank p < 0.05 was statistically significant.

### Methylation and expression analysis

The methylation levels of ARNTL2 in LUAD and paracancerous normal tissues were then assessed using the UALCAN (http://ualcan.path.uab.edu/analysis.html) and MEXPRESS (https://mexpress.be/old/mexpress.php). The survival analysis of methylation data (CpG islands) was performed using the MethSurv database (https://biit.cs.ut.ee/methsurv/).

### Immune infiltration analysis in LUAD

To study the relationship between ARNTL2 expression and tumor-infiltrating immune cells, we used TIMER (https://cistrome.shinyapps.io/timer/), an online tool for analyzing and visualizing the link between immune infiltration levels and various cancer.

### Cells and cell culture conditions

The normal epithelial lung cell BASE-2B and lung cancer cell lines BASE-2B, H1299, PC9, and A549 were obtained from the Cell Bank of the Chinese Academy of Sciences (Shanghai, China). The BEAS-2B and A549 cells were cultured at 37℃ in a 5% CO2 incubator in Dulbecco’s modified Eagle’s medium (DMEM, HyClone, Logan City, UT) with 10% fetal bovine serum (FBS; Gibco, Carlsbad, CA), penicillin (100 IU/ml), and streptomycin sulfate (100 μg/ml). The H1299 and PC9 cells were maintained in Roswell Park Memorial Institute (RPMI) 1640 medium (HyClone) with the same supplementation in the same incubator.

### Transfection

The H1299 cells with the highest ITGB1-DT expression were selected for further experiments. The ITGB1-DT siRNA and hsa-miR-30b-3p mimic were designed and synthesized from RiboBio Co. Ltd (Guangzhou, China). And it was transfected using riboFECT^TM^CP Reagent according to the manufacturer’s instruction.

### Real-time quantitative PCR

Total cell RNA was extracted by using Trizol reagent (Invitrogen, CA, USA). The reverse transcription and PCR process was performed according to the manufacturer’s instructions (Applied Biosystem, ThermoFisher Scientific, Shanghai, China). The whole procedure of the experiment was carried out according to our previous study [38]. The mRNA primers were designed and synthesized by the Sangon Biotech Co. Ltd. (Shanghai, China). And the miRNA primers were synthesized by RiboBio Co. Ltd (Guangzhou, China). The primer sequences were supplemented in Additional file 1: Table S4.

### CCK-8 and transwell assay

The proliferation of H1299 cells were detected by performing the CCK-8 assay according to the manufacturer’s instruction. H1299 cells (1 × 10^3^) were plated in 96-well plates and treated with 10 μl of CCK-8 solution for the next 24, 48, and 72 h. Then, the proliferation of cells was assessed at 450 nm.

Using transwell chamber (Corning, NY, USA) to conduct the invasion assay. After culturing for 24 h, the cells on the upper surfaces of chambers were wiped with cotton swabs, and the cells on the lower surfaces were fixed with 4% paraformaldehyde for 10 min, followed by stained with crystal violet (Beijing Solarbio Science and Technology Co., Ltd) for 5 min. Then, the cells were photographed and counted under a microscope.

### Colony formation assay

The H1299 cells were seeded in to the 6-well plates at the density of 1000 cells/well. Then, cells were cultured for 7 days and stained with 0.1% crystal violet (Beijing Solarbio Science and Technology Co., Ltd) for 10 min. And the colony numbers were counted using Fiji software [39].

### Statistical analysis

The differences between two sets of TCGA data were assessed utilizing Wilcoxon Signed rank test, Mann–Whitney U test. An independent t-test was utilized to evaluate the difference between two groups of GEO data. And p < 0.05 was considered statistically significant.

## Results

### The role and expression of SLC2A1 in LUAD

**Fig. 2:**
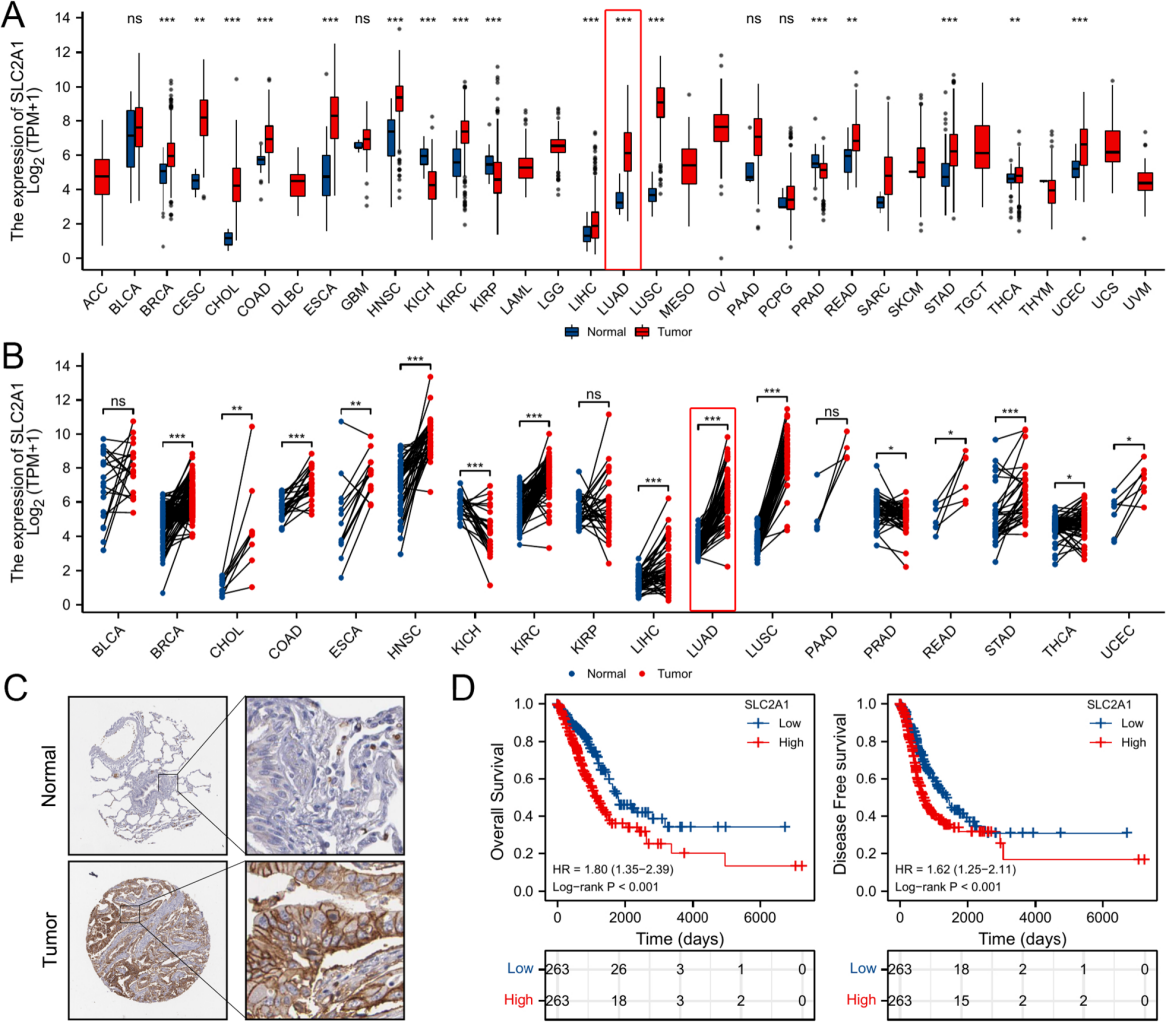
Role of SLC2A1 in LUAD. **A** The expression of SLC2A1 in unpaired pan-cancer tissues (* < 0.05, ** < 0.01, *** < 0.001). **B** The expression of SLC2A1 in paired pan-cancer tissues (* < 0.05, ** < 0.01, *** < 0.001). **C** The immunohistochemistry of SLC2A1 in LUAD. **D** The overall survival and disease-free survival of SLC2A1 in LUAD patients (OS: p < 0.001, HR: 1.80 (1.35–2.39); DFS: p < 0.001, HR: 1.62 (1.25–2.11))

The expression level of SLC2A1 is frequently upregulated in multiple cancers, including LUAD (Fig. 2A, B). Based on the Human Protein Atlas database (https://www.proteinatlas.org/), the immunohistochemistry (IHC) results showed that SLC2A1 was overexpressed in LUAD tissues, but low expressed in the normal lung tissues (Fig. 2C). According to the Human Protein Atlas database, the SCL2A1 is frequently at a low expression level in various normal tissues (Additional file 1: Fig. S1). SLC2A1 expression and clinical data obtained from the TCGA database were used for survival analysis. And the results pointed out that the patients with high SLC2A1 expression have a poor prognosis (HR = 1.80, 95% CI: (1.35–2.39), p < 0.001) and a high recurrence rate (HR = 1.62, 95% CI: (1.25–2.11), p < 0.001) (Fig. 2D). According to the ranking of SLC2A1 expression, the first 25% and the last 25% samples were selected for survival analysis, and the results are shown in Additional file 1: Fig. S1. Besides, we also performed the survival analysis of GLUT3, and the results show that only the SLC2A1 is related to the prognosis of LUAD (Additional file 1: Fig. S1). In view of the role of SLC2A1 in LUAD, we speculated that the differential genes obtained by differential analysis based on SLC2A1 expression grouping also have the value of the in-depth study. Moreover, it is possible to find potential therapeutic targets that affect the progression of LUAD.

### Analysis of DElncRNAs, DEmiRNAs, and DEmRNAs

**Fig. 3:**
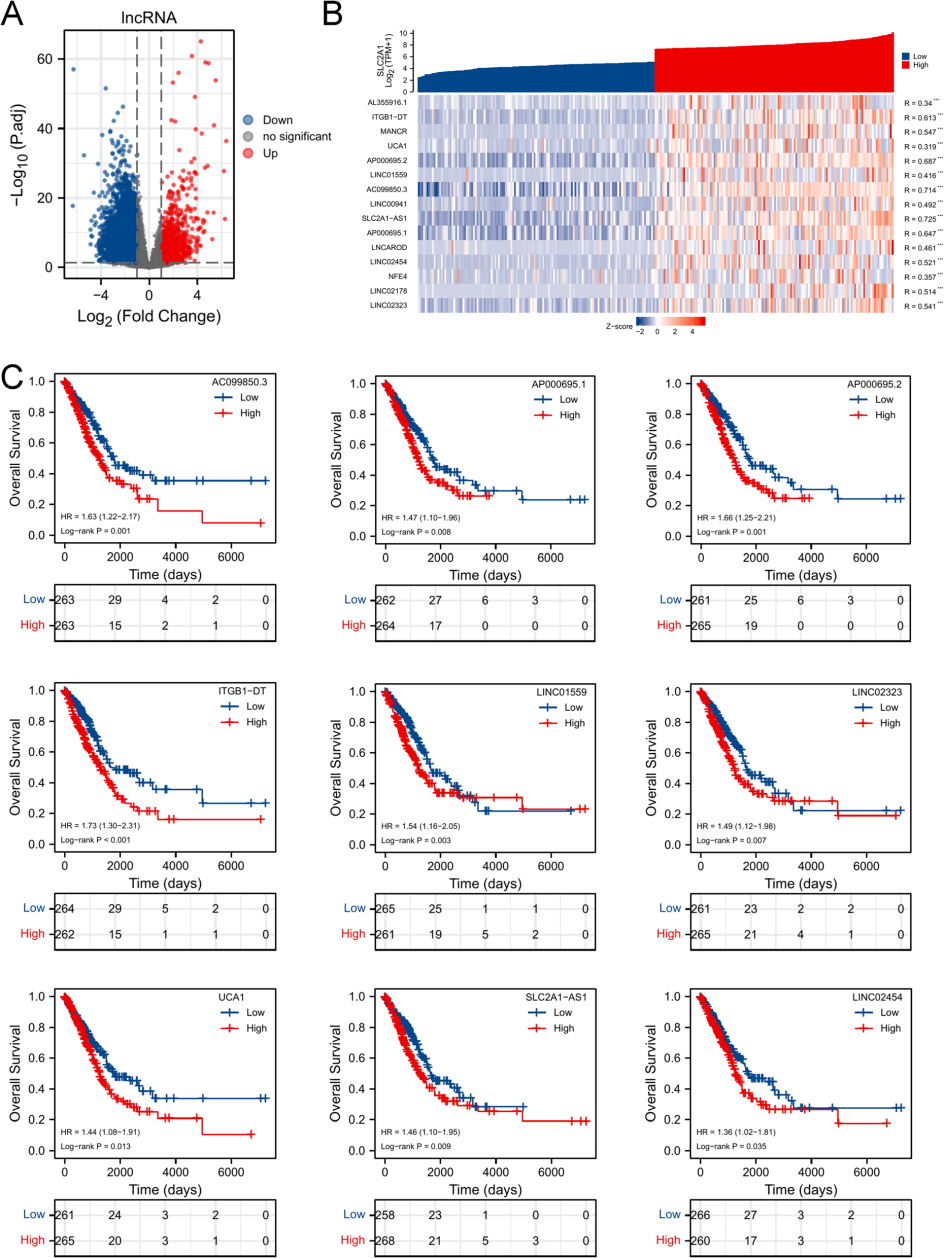
Analysis of DElncRNAs. **A** The volcano map of DElncRNAs based on the SLC2A1expression (|logFC|> 1, p.adj < 0.05). **B** The co-expression heatmap of top 15 upregulated lncRNAs and SLC2A1 (* < 0.05, ** < 0.01, *** < 0.001). **C** Suvival curve of lncRNAs (log-rank p < 0.05)

According to the ranking of SLC2A1 expression, we chose the first 25% samples (cut-off value: SLC2A1 (TPM) = 154.6060991) and the last 25% samples (cut-off value: SLC2A1 (TPM) = 32.56593626) for differential lncRNA analysis. Then, 778 upregulated and 3298 downregulated lncRNAs were visualized by volcano plot (Fig. 3A). Then, we selected the top 15 upregulated lncRNAs for gene co-expression analysis, and we found a strong correlation between the top 15 upregulated lncRNAs and SLC2A1 (Fig. 3B). Survival analysis suggested that 9 of 15 upregulated lncRNAs were associated with the poor prognosis of LUAD (Fig. 3C, Additional file 1: Fig. S2).

**Fig. 4:**
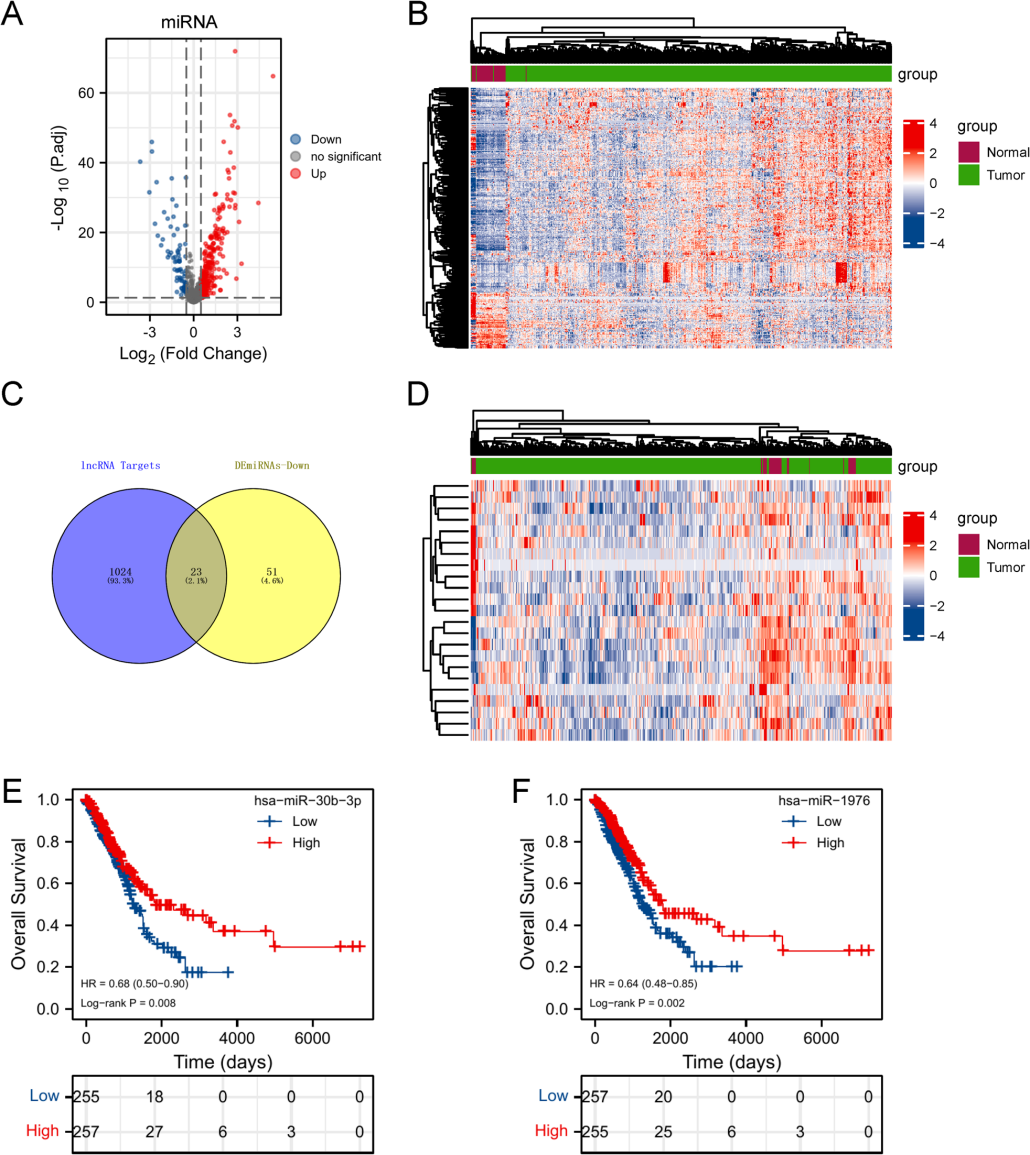
Analysis of DEmiRNAs. **A** The volcano map of DEmiRNAs between tumor and normal tissues (|logFC|> 0.5, p.adj < 0.05). **B** The heatmap of DEmiRNAs. **C** 23 overlapping miRNAs between lncRNA targets and downregulated DEmiRNAs. **D** The heatmap of 23 overlapping miRNAs. **E**, **F** The significant survival curves of miRNA (log-rank p < 0.05)

The mature miRNA data were downloaded from the TCGA database. 268 upregulated and 74 downregulated miRNAs in LUAD tissues were visualized by volcano plot, and the expression level was shown in the heatmap (Fig. 4A, B). We first imported the 9 lncRNAs to the Tarbase v8.0 database (http://carolina.imis.athena-innovation.gr/diana_tools/web/index.php?r=tarbasev8%2Findex) for predicting the potential target miRNAs. And 23 miRNAs were obtained by taking the intersection of lncRNAs’ target miRNAs and DEmiRNAs (Fig. 4C, Table 1). The expression level of 23 miRNAs was visualized by heatmap (Fig. 4D). Ultimately, 2 lncRNAs (SLC2A1-AS1 and ITGB1-DT) and 2 miRNAs (hsa-miR-30b-3p (HR = 0.68, 95% CI: 0.50–0.90, p = 0.008) and hsa-miR-1976 (HR = 0.64, 95% CI: 0.48–0.85, p = 0.002)) related to the outcome of LUAD were obtained by performing the survival analyses. (Fig. 4E, F, Additional file 1: Fig. S3).

**Table 1:** The predicted miRNAs of differentially expressed lncRNAs

lncRNA	miRNA
ENSG00000180861 (LINC01559)	hsa-miR-30b-3p, hsa-miR-223-3p, hsa-miR-423-5p, hsa-miR-520a-3p, hsa-miR-138-5p, hsa-miR-2110, hsa-miR-3154, hsa-miR-26a-5p
ENSG00000214049 (UCA1)	hsa-miR-486-3p, hsa-miR-423-5p, hsa-miR-3614-5p hsa-miR-143-3p, hsa-miR-125b-2-3p, hsa-miR-1-3p, hsa-miR-206, hsa-miR-374a-5p, hsa-miR-27a-5p, hsa-miR-340-5p, hsa-miR-184
ENSG00000227533 (SLC2A1-AS1)	hsa-miR-1976, hsa-miR-204-5p, hsa-miR-34b-3p, hsa-miR-3614-5p
ENSG00000229656 (ITGB1-DT)	hsa-miR-30b-3p
ENSG00000233818 (AP000695.2)	hsa-miR-145-3p, hsa-miR-144-3p

**Fig. 5:**
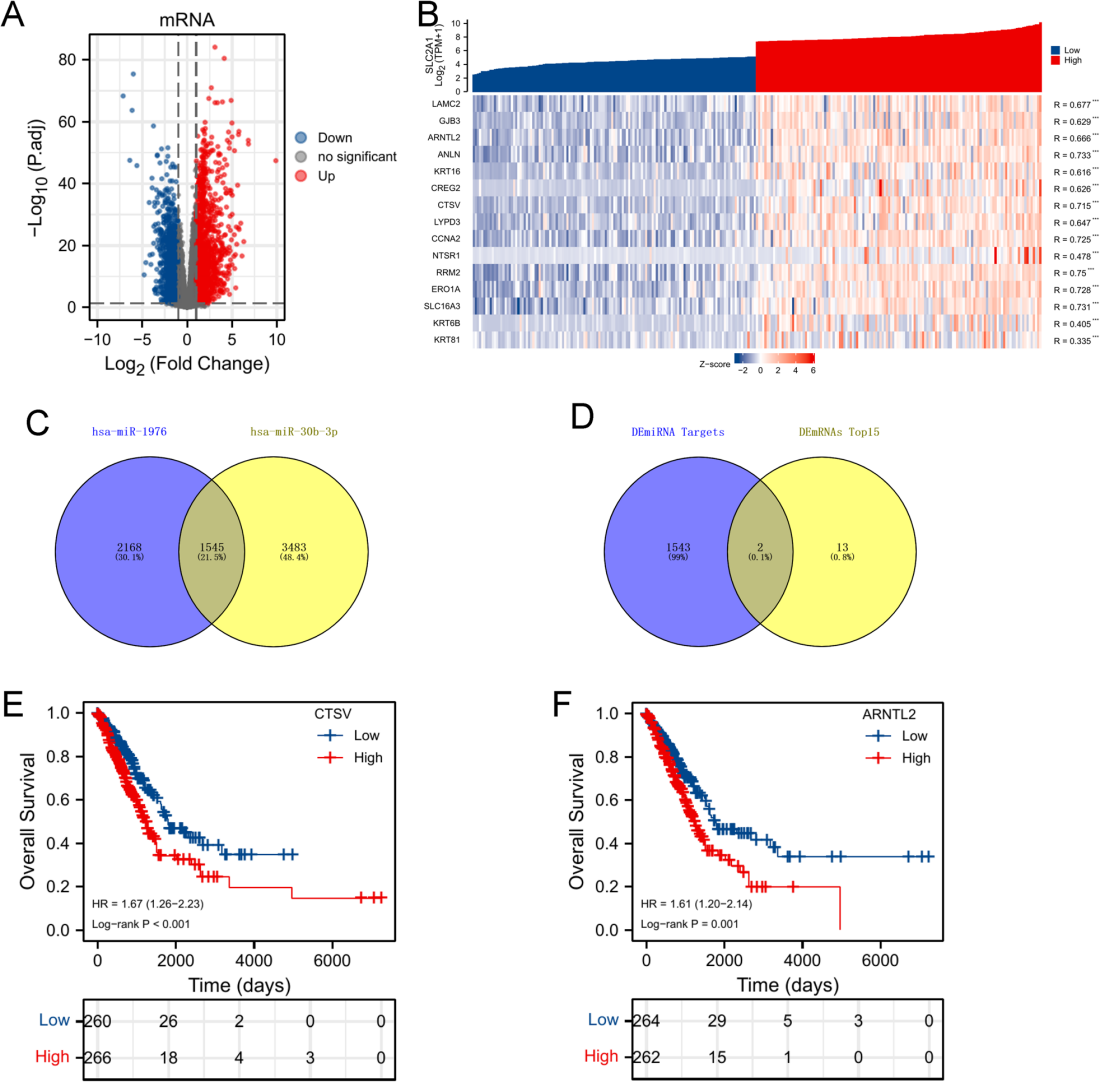
Analysis of DEmRNAs. **A** The volcano map of DEmRNAs based on the SLC2A1 expression (|logFC|> 1, p.adj < 0.05). **B** The co-expression heatmap of top 15 upregulated mRNAs and SLC2A1 (* < 0.05, ** < 0.01, *** < 0.001). **C** The overlapping targets between hsa-miR-1976 and hsa-miR-30b-3p. **D** The final overlapping mRNAs between DEmiRNAs targets and top15 DEmRNAs. **E**, **F** The significant survival curves of final hub mRNAs (log-rank p < 0.05)

Furthermore, 1535 upregulated and 1504 downregulated mRNAs were confirmed between the high SLC2A1 expression group and the low SLC2A1 expression group (Fig. 5A). Then the top 15 upregulated mRNAs were selected for the gene co-expression analysis (Fig. 5B). And the hsa-miR-30b-3p and hsa-miR-1976 were imported to the miRWalk 2.0 database for predicting the potential mRNA targets. By taking the intersection between miRNAs’ targets and top 15 upregulated DEmRNAs, 2 DEmRNAs (CTSV and ARNTL2) were ultimately identified as oncogenes using survival analysis in LUAD tissues (Fig. 5C, D). The results of survival analysis showed that CTSV (HR = 1.67, 95% CI:1.26–2.23, p < 0.001) and ARNTL2 (HR = 1.61, 95% CI: 1.20–2.14, p = 0.001) predicted the poor prognosis of LUAD (Fig. 5E, F). This series of analyses helped us identify several LUAD-related prognostic genes.

### Correlation between DElncRNAs, DEmiRNAs, and DEmRNAs

**Fig. 6:**
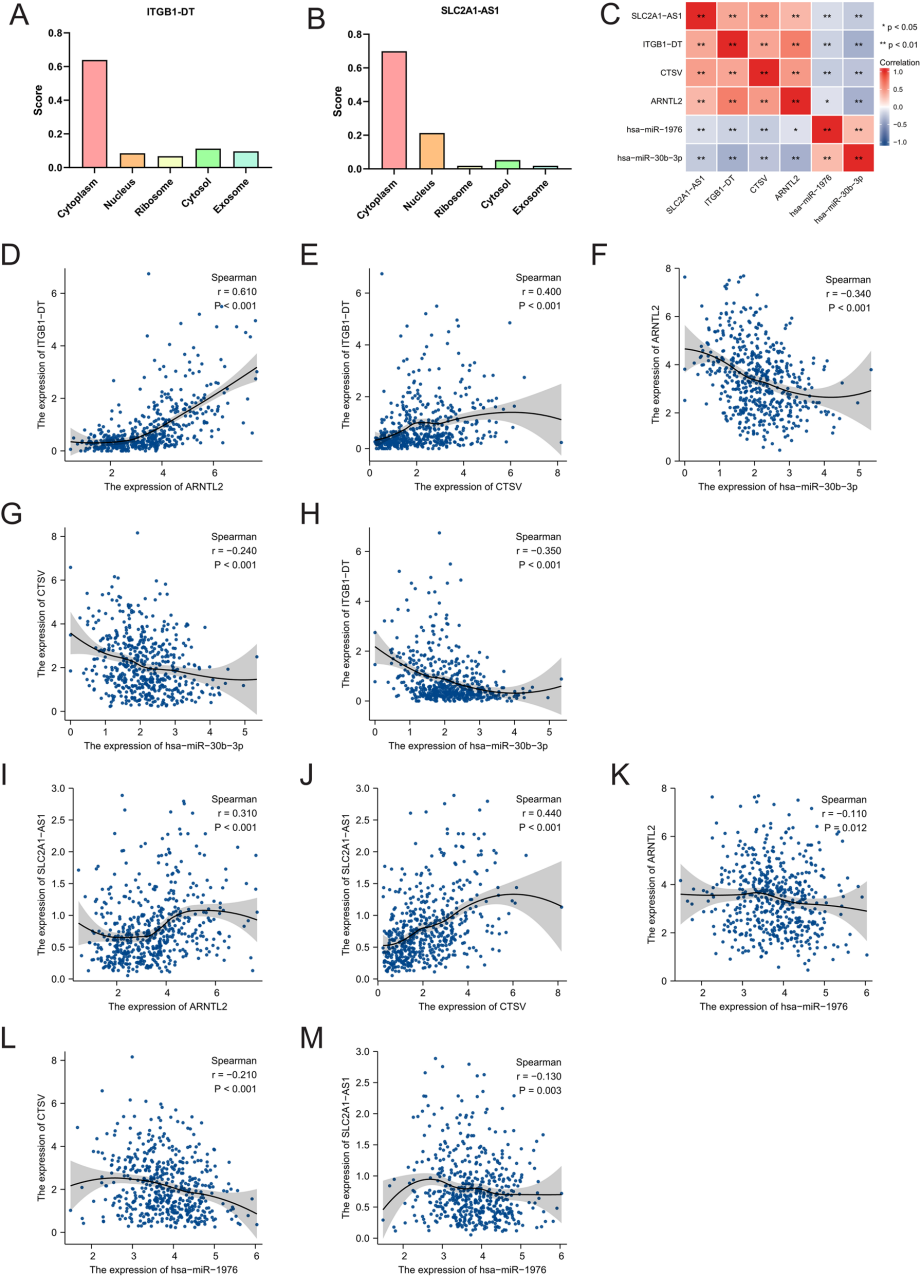
Correlation between DElncRNAs, DEmiRNAs, and DEmRNAs. **A**, **B** The cellular localization of ITGB1-DT and SLC2A1-AS. **C** The correlation heatmap of hub genes. **D**–**M** Correlation analysis between the DElncRNAs, DEmiRNAs, and DEmRNAs

Furthermore, considering that there may be a link between the cellular localization of lncRNAs and the mechanism of action, we predicted the subcellular localization of the three DElncRNAs by uploading the lncRNA sequences obtained from the LNCipedia database (https://lncipedia.org/) (Additional file 1: Table S1) to the lncLocator analysis tool (http://www.csbio.sjtu.edu.cn/bioinf/lncLocator/). We found that lncRNA SLC2A1-AS1 and ITGB1-DT were mainly located in the cytoplasm (Fig. 6A, B).

We first performed the Shapiro–Wilk normality test and found that some of the variables did not meet the normal distribution, so we utilized the Spearman correlation analysis to analyze the correlation between hub genes. For the correlation analysis results (Fig. 6C), we found that there is a positive relation between lncRNA ITGB1-DT and ARNTL2 (R = 0.610, p < 0.0001, Fig. 6D) and CTSV (R = 0.400, p < 0.0001, Fig. 6E), while there is a negative relation between hsa-miR-30b-3p and lncRNA ITGB1-DT (R = − 0.350, p < 0.001, Fig. 6H), ARNTL2 (R = − 0.340, p < 0.001, Fig. 6F) and CTSV (R = − 0.240, p < 0.001, Fig. 6G).

Meanwhile, results showed that there is a negative association between hsa-miR-1976 and ARNTL2 (R = − 0.110, p = 0.012, Fig. 6K), CTSV (R = − 0.210, p < 0.001, Fig. 6L) and lncRNA SLC2A1-AS1 (R = − 0.130, p = 0.003, Fig. 6M), while there is a positive correlation between lncRNA SLC2A1-AS and ARNTL2 (R = 0.310, p < 0.001, Fig. 6I) and CTSV (R = 0.440, p < 0.001, Fig. 6J). Bioinformatics analyses were performed to predict that there are exiting binding sites between the lncRNA, miRNA, and mRNA. Also, the results of correlation analyses confirmed the previous hypothesis. Therefore, the next step of the study can be carried on based on these analysis results.

### Methylation analysis of ARNTL2

**Fig. 7:**
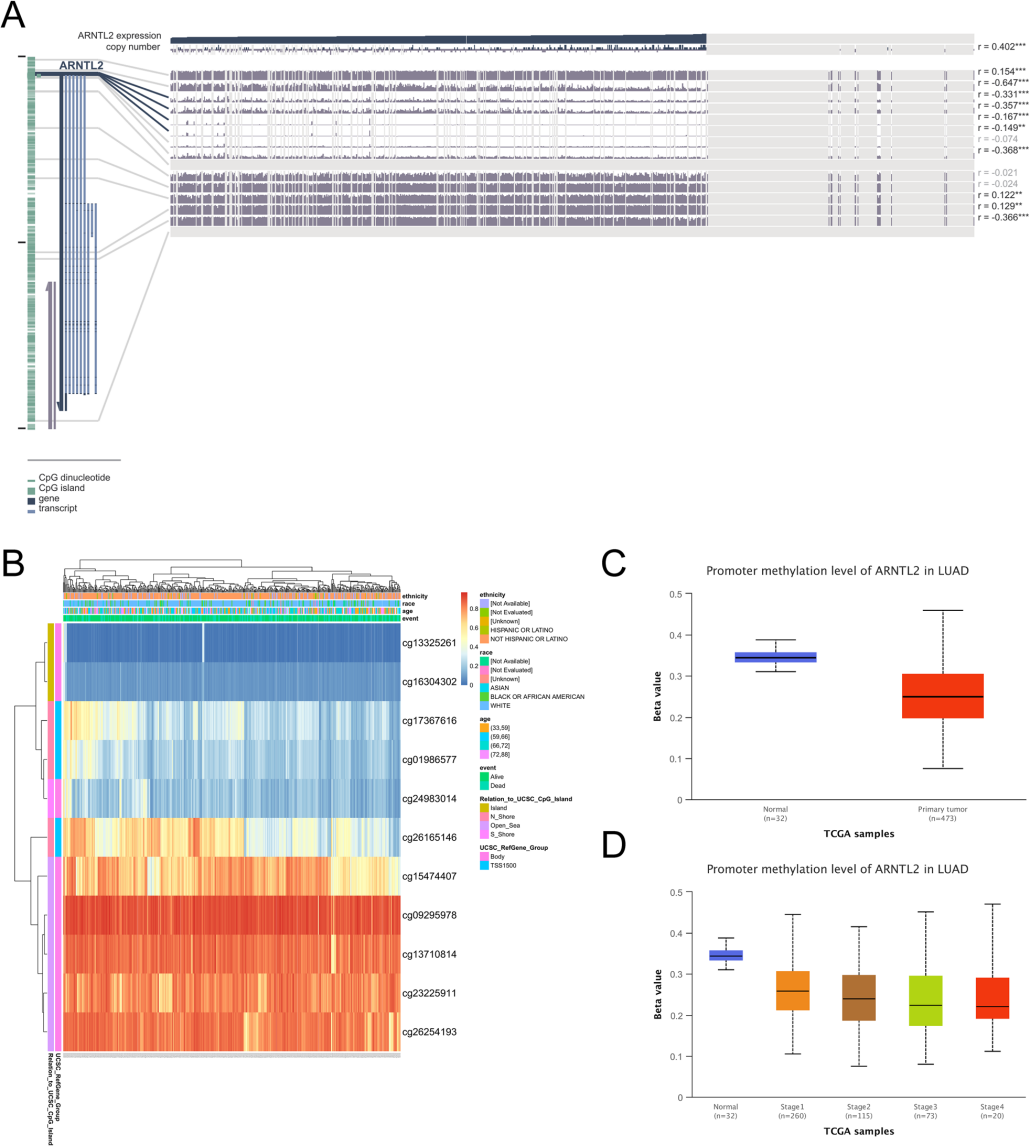
Methylation analysis of ARNTL2. **A** The correlation between ANRTL2 gene expression and methylation level. **B** The expression level of different CpG island. **C**, **D** The promoter methylation level of ARNTL2 in LUAD patients at different stages were visualized by using UALCAN database

Then, the comprehensive analysis of ROC curve results revealed that ITGB1-DT/miR-30b-3p/ARNTL2 has a significant prognostic value in LUAD. The area under the curve (AUC) of ITGB1-DT, hsa-miR-30b-3p, and ARNTL2 was 0.828, 0.718, and 0.847, respectively (Additional file 1: Fig. S4). In recent years, many studies have found that alterations of DNA methylation in tumors have been identified as the most promising targets for developing powerful diagnostic, prognostic, and predictive biomarkers of disease onset [40]. Therefore, we investigated the correlation between ARNTL2 levels and methylation status using various methods for exploring the mechanism of abnormal ARNTL2 expression in LUAD tissues, We first analyzed the correlation between three methyltransferases and ARNTL2 levels in LUAD tissues. The results showed that DNMT1 (R = 0.190, p < 0.001) and DNMT3B (R = 0.210, p < 0.001) were correspondingly upregulated with the elevated expression of ARNTL2 (Additional file 1: Fig. S4). Furthermore, through the MEXPRESS database, 4 promoter methylation sites (cg01986577, cg17367616, cg22952210, and cg13325261) were identified in the DNA sequence of ARNTL2, and the methylation level is negatively correlated with ARNTL2 expression (Fig. 7A). The CpG site expression was shown in Fig. 7B. The probe ID and sequence around CpG island were shown in Additional file 1: Table S2. The promoter methylation level of ARNTL2 was lower in LUAD tumor tissues than that in normal tissues (p < 0.001) and was related to the advanced stage (p < 0.01) (Fig. 7C, D). All the findings indicated that the abnormal expression levels of ARNTL2 may be due to altered methylation levels, which also implies that altered promoter methylation levels of ARNTL2 may be partially responsible for the development of LUAD.

### Correlation between ARNTL2 expression and immune infiltration in LUAD

**Fig. 8:**
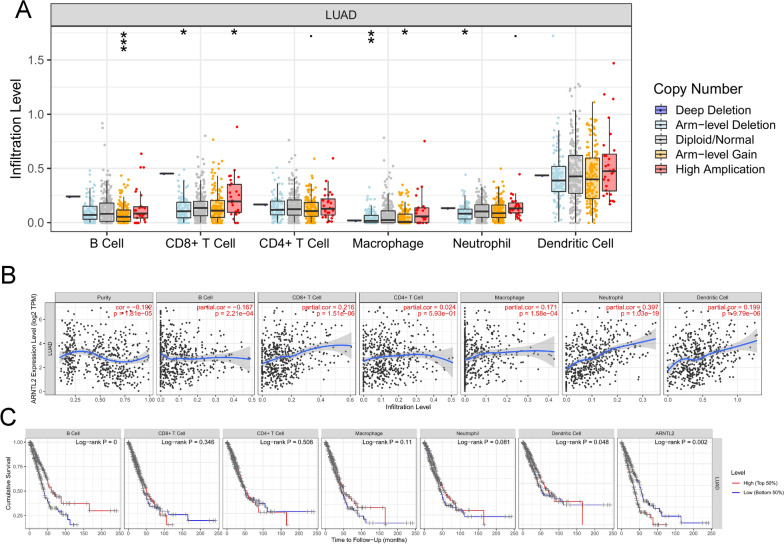
Correlation between ARNTL2 expression and immune infiltration in LUAD. **A** Correlation between ARNTL2 gene copy number and infiltration level. **B** Correlation between ARNTL2 expression and different immune cells infiltration level. **C** The overall survival of different immune cells infiltration level in LUAD

Earlier studies have reported that immune cells (including B cells, macrophages, CD8( +) T cells, and so on) are closely related to lung cancer [41–43]. Moreover, it was reported that tumor purity as a confounding factor affects gene expression and DNA methylation levels, and then copy number affects gene expression levels, which in turn is related to the immune cell infiltration levels [44]. Therefore, by using TIMER database, we carried on the following studies to investigate whether there is a relationship between ARNTL2 expression and the level of immune infiltration. As shown in Fig. 8A, the “SCNA” module analysis revealed that in LUAD, immune cells including B cell, CD8 + T cell, macrophage, and neutrophil, appeared to be associated with ARNTL2 gene copy numbers. Moreover, the correlation between promoter methylation level and immunue infiltration was showed in Additional file 1: Fig. S5. From the results of “GENE” module analysis, ARNTL2 expression is not only significantly related to tumor purity, but it is also closely associated with immune infiltration levels of B cell, CD8 + cell, macrophage, neutrophil, and dendritic cell in LUAD (Fig. 8B). Ultimately, the effect of immune infiltration on LUAD prognosis was further determined. And the results revealed that the low immune infiltration levels of B cell and the dendritic cell were related to the poor prognosis of LUAD (Fig. 8C).

### Functional enrichment analysis of ARNTL2

The GSEA was conducted to further explore the potential function and pathway of ARNTL2. The results of GSEA revealed that the samples of highly expressed ARNTL2 were mainly enriched in “olfactory transduction”, “cytokine-cytokine receptor interaction”, “systemic lupus erythematosus”, “natural killer cell-mediated cytotoxicity” and “cell cycle” (Additional file 1: Fig. S6A), whereas the samples of lowly expressed ARNTL2 were mainly enriched in “GNRH signaling pathway”, “Arachidonic acid metabolism”, and “alpha linolenic acid metabolism” (Additional file 1: Fig. S6B). Although the “olfactory transduction” and “systemic lupus erythematosus” are not our main research subjects, the “cytokine-cytokine receptor interaction” and “natural killer cell-mediated cytotoxicity” and “cell cycle” as the most common signaling pathways play an important roles in our study. In the meantime, the enriched immune-related pathways coincide with our immune infiltration studies, suggesting that ARNTL2 may induce the development of LUAD by altering the immune microenvironment. The GO and KEGG pathway analysis of top 200 ARNTL2-related genes revealed that the most significantly enriched pathways are “Central carbon metabolism in cancer”, “Focal adhesion”, “ECM-receptor interaction”, “Small cell lung cancer”, and “Regulation of actin cytoskeleton”. And the KEGG results also reflects the function of ARNTL2 and its related genes in cancer, even in LUAD. Besides, the GO BP terms of ARNTL2-related genes are mainly “hemidesmosome assembly”, “cell-substrate junction assembly”, “cell junction assembly”, “cell junction organization”, and “cell-substrate adhesion”; the GO CC terms are mainly “focal adhesion”, “cell-substrate adherens junction”, “cell-substrate junction”, “cell–cell junction”, and “lamellipodium”; the GO MF terms are mainly “cell adhesion molecule binding”, “actin-binding”, “cadherin binding”, “actin filament binding”, and “structural constituent of cytoskeleton” (Additional file 1: Fig. S6C, D).

### Validation of gene expression in LUAD

**Fig. 9:**
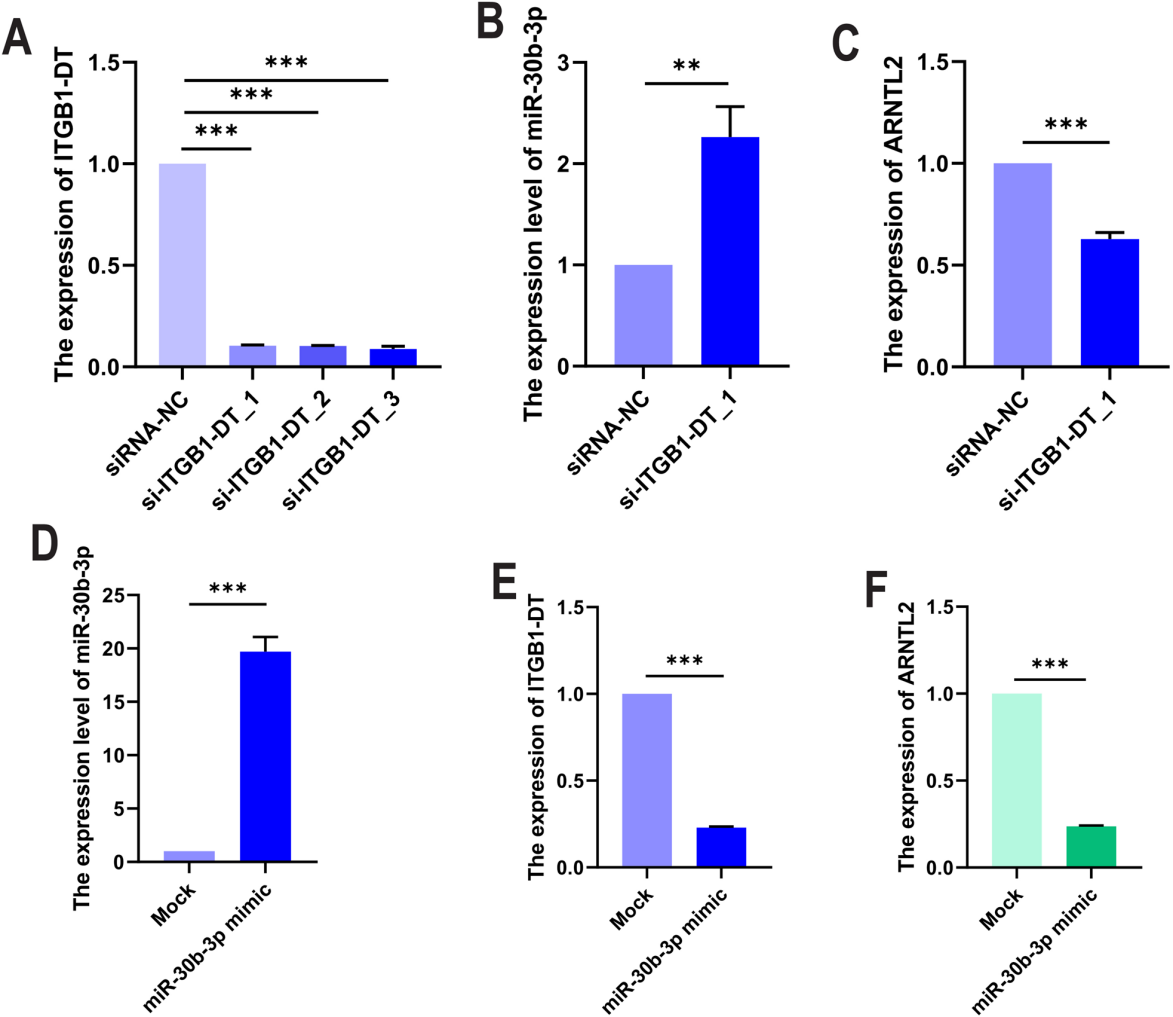
Knockdown of ITGB1-DT regulates the expression of hsa-miR-30b-3p and ARNTL2. **A** The interference of ITGB1-DT expression in H1299 cells. **B** and **C** The expression of miR-30b-3p and ARNTL2 in H1299-si-ITGB1-DT_1 cell. **D** The miR-30b-3p expression in H1299-miR-30b-3p mimic cells. **E** and **F** The ITGB1-DT and ARNTL2 expression in H1299-miR-30b-3p mimic cells

Through the Cancer Cell Line Encyclopedia (CCLE) database (https://portals.broadinstitute.org/ccle), ARNTL2 was frequently highly expressed in lung cancer cell lines (Additional file 1: Fig. S7A). Moreover, we downloaded the GSE43458 microarray from GEO database for the difference analysis of ARNTL2 in 110 LUAD patients (consisting of 30 normal tissues and 80 tumor tissues). Interestingly, the analysis result was consistent with our TCGA findings (Additional file 1: Fig. S7B). Furthermore, we also performed the qRT-PCR assay to detect the expression level of ITGB1-DT, has-miR-30b-3p, and ARNTL2 in LUAD cell lines, the results also suggested that the ITGB1-DT and ARNTL2 expression were significantly increased in the LUAD cell lines (A549, H1299, and PC9), while the has-miR-30b-3p expression was higher in normal lung epithelial cell line (BEAS-2B) than that in LUAD cell lines (Additional file 1: Fig. S7C–E). In order to assess the association between lncRNA, miRNA, and mRNA, we transfected the ITGB1-DT-siRNAs and hsa-miR-30b-3p mimic into the H1299 cell line to construct H1299-siITGB1-DT cells for further experiments. Figure 9A presents stable knockdown of ITGB1-DT expression in H1299 cell line. Interestingly, the results of qRT-PCR was observed that ARNTL2 expression of H1299-si-ITGB1-DT cells correspondingly decreased compared to the control group (Fig. 9C), but the hsa-miR-30b-3p expression in H1299-siITGB1-DT cells unexpectedly increased (Fig. 9B). Overall, these results suggested that ITGB1-DT regulates the expression of hsa-miR-30b-3p and ARNTL2 in LUAD. Instead, we also detected the expression of ITGB1-DT and ARNTL2 in H1299-hsa-miR-30b-3p mimic cells. The stable upregulation of hsa-miR-30b-3p expression in H1299-hsa-miR-30b-3p mimic cells was confirmed by qRT-PCR (Fig. 9D). Figure 9E, F shows that there is a significant decrease in the expression of ITGB1-DT and ARNTL2 after the upregulation of hsa-miR-30b-3p. These results together elucidated that hsa-miR-30b-3p can also regulate the expression of ITGB1-DT and ARNTL2.

### Knocdown of ITGB1-DT inhibits the LUAD progression

**Fig. 10:**
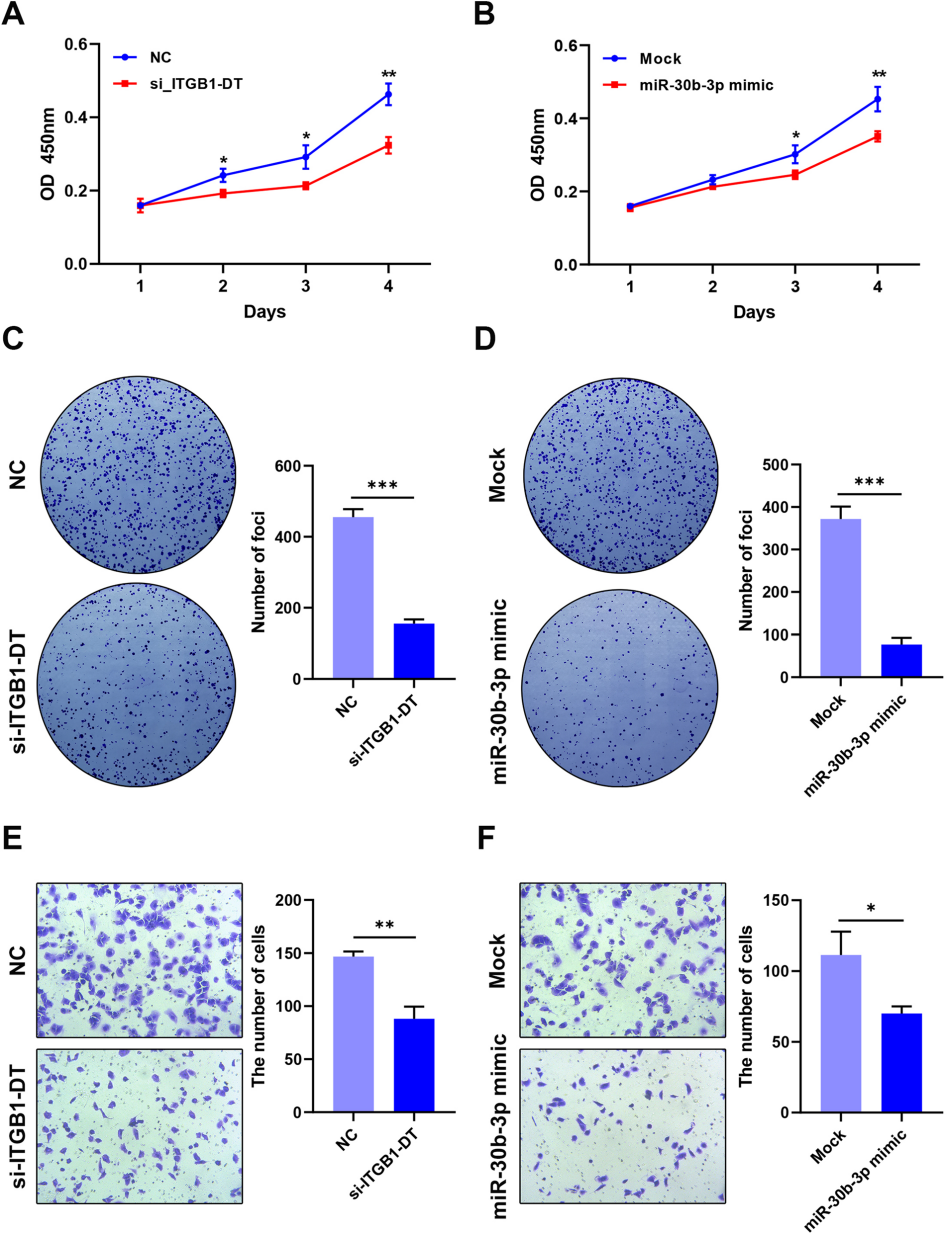
Knockdown of ITGB1-DT inhibits the LUAD progression. **A**–**D** Cell viability was determined in H1299-si-ITGB1-DT and hsa-miR-30b-3p mimic cell using CCK-8 and colony formation assays (* p < 0.05, ** p < 0.01). **E** and **F**) Invasive ability was detected in H1299-si-ITGB1-DT and hsa-miR-30b-3p mimic cell using transwell assays (* p < 0.05, ** p < 0.01)

To explore the role of ITGB1-DT in LUAD, we performed the corresponding cellular function experiments. The proliferation of H1299 cells was detected through the CCK-8 assay. And the results showed that the proliferation of H1299-siITGB1-DT cells was significantly inhibited compared with the negative control group (Fig. 10A). Moreover, the proliferation of cells transfected with hsa-miR-30b-3p mimic was also significantly inhibited compared to the mock group (Fig. 10B). The colony formation assay was also performed to investigate the proliferation of H1299 cells. And results showed that the downregulation of ITG1-DT or upregulation of hsa-miR-30b-3p impair the proliferative ability of H1299 cancer cells (Fig. 10C, D).

Afterwards, the invasive ability of H1299 cells subjected to different treatments was examined by transwell assays. As shown in Fig. 10C, the numer of cells penetrating the chamber in siITGB1-DT group is significantly less than that in negative control group, which implied that interference with the expression of ITGB1-DT affects the invasive ability of the H1299 cells. Interestingly, we observed the same results in the group transfected with has-miR-30b-3p mimic and its corresponding control group (Fig. 10D).

### Prognostic analysis in LUAD

To better determine the prognostic significance of hub genes, we established the Cox regression analysis model to assess the survival-related feature. In univariate Cox regression analysis, we found that pathologic stage, ITGB1-DT, has-miR-30b-3p, and ARNTL2 as prognostic factors were significantly associated with overall survival in LUAD patients (Additional file 1: Fig. S8A–C). A prediction model was visualized by nomogram (Additional file 1: Fig. S8D–F).

### Prediction of drugs

Clinically, the patients still prefer to be treated mainly with drugs. Therefore, several drugs related to ARNTL2 gene were predicted using the DSigDB database (http://tanlab.ucdenver.edu/DSigDB). As shown in Additional file 1: Figure S9 and Table S3, Panobinostat (p = 0.0038), Raloxifene (p = 0.008), and Diltiazem (p = 0.0182), have potential to improve the adverse prognosis of LUAD.

## Discussion

Lung cancer is one of the deadliest malignant tumors worldwide due to its poor prognosis and high aggressiveness [45]. Among them, lung adenocarcinoma (LUAD) has become the most frequent histological type of malignant lung cancer during the past decades [46]. In recent years, although the prognosis of LUAD has improved significantly with the development of medical technology, the average 5-year survival rate is still only about 15% [47, 48]. Meanwhile, the mechanisms of LUAD tumorigenesis remain unclear. Therefore, to improve the outcome and decrease the mortality, novel biomarkers and treatments are urgently needed to be explored. Previous reports indicated that lncRNA, as a sponge of miRNA, can regulate the translation process of gene expression in LUAD [49]. For example, Zhao et. al confirmed that lncRNA HOXA11-AS promotes cisplatin resistance of LUAD cells via regulating miR-454-3p/STAT3 [50]. However, the mechanisms of the lncRNA-mediated ceRNA regulatory network still need further investigation. In the meantime, as the mechanism of LUAD is studied in-depth, it can also help us better understand the pathogenesis of LUAD and seek new treatments.

In our study, we aimed to develop a SLC2A1-related ceRNA network that is related to the outcome of LUAD. It is well known that cancer cells consume more glucose for energy through the “Warburg effect” [51]. So, when SLC2A1 is abnormally activated in the plasma membrane of tumor cells, it can transport large amounts of glucose from the extracellular to the mitochondria, thus providing energy to the cancer cells [52]. According to previous research, SLC2A1 appears to serve as an oncogene in a variety of malignancies, including lung cancer [31, 53, 54], pancreatic cancer [28], oral cancer [55], and so on. Meanwhile, SLC2A1 was reported to play a role as a drug target for cancer therapy. It mainly affects the glycolytic pathway of cancer cells, and the inactivation of SLC2A1 eventually leads to apoptosis in vitro and in vivo [53]. In light of the critical role of SLC2A1 in the progression of LUAD, it is of interest to explore corresponding differential genes based on SLC2A1 expression levels. Likewise, the results of survival analysis, IHC, and copy number variation analysis in our study are consistent with previous research. Based on the SLC2A1 expression, we divided all the LUAD patients into SLC2A1^high^ and SLC2A1^low^ groups. After performing the difference analysis of lncRNAs, miRNAs, and mRNAs, we finally obtained 2 lncRNAs (ITGB1-DT and SLC2A1-AS), 2 miRNAs (hsa-miR-30b-3p and hsa-miR-1976), and 2 mRNAs (CSTV and ARNTL2). Then, the ROC curve of six genes indicated that ITGB1-DT/hsa-miR-30b-3p/ARNTL2 axis may be related to the progression of LUAD. Moreover, the further analysis of ITGB1-DT, hsa-miR-30b-3p, and ARNTL2 suggested that they are all significantly associated with the outcome of LUAD.

Next, the roles of ITGB1-DT, miR-30b-3p, and ARNTL2 were found to be involved in cancers. In line with previous study, our sublocalization analysis of lncRNAs provided a good basis for the extend further research. It was reported that cytoplasmic lncRNAs can attenuate the biological function of interacted miRNAs [56]. And many studies have been elucidated that cytoplasmic lncRNA could bind not only miRNAs but also mRNAs. For example, MRCCAT1 promotes metastasis of clear cell renal cell carcinoma via inhibiting NPR3 and activating p38-MAPK signaling [57]. Moreover, LDLRAD4-AS1 promotes metastasis through the downregulated of LDLRAD4, which predicts a poor prognosis in colorectal cancer [58]. Thus, the key role of cytoplasmic lncRNAs in tumors is unquestionable. And the latest reports have elucidated that elevated ITGB1-DT promotes LUAD progression by forming a positive feedback loop with ITGB1/Wnt/β-Catenin/MYC [59].

Meanwhile, growing evidence showed that miRNAs existed in various tissues and cells. It is actually affected different cancer progression by regulating a wide range of cancer-related genes [60]. Besides, it was reported that miRNAs have a great potential for cancer diagnosis [61]. Such as, Lisa Kinget et.al found that the hsa-miR-30b-3p could suppress the development of bone metastasis in clear-cell renal cell carcinoma [62]. And the downregulation of hsa-miR-30b-3p leads to the poor prognosis of gastric cancer [63]. Besides, miR-30b-3p affects the outcome of ovarian cancer, hepatocellular, and so on [64–66].

The role of mRNA in disease processes is even more indisputable, and basically most of the articles cannot be separated from its scope. Some research have suggested that ARNTL2 served as an oncogene that could enhance the development of various cancers. Zhifang Wang et.al found that miR-26a-5p suppressed the pancreatic ductal adenocarcinoma (PDAC) progression by targeting ARNTL2, which means that ARNTl2 acted as an oncogene to regulate PDAC growth [67]. Furthermore, the study of Min Lu et.al also suggested that ARNTL2 could suppress colon carcinoma cell proliferation and migration via SMOC-EMT through inactivating the PI3K/AKT signaling pathway [68]. And in our study, ITGB1-DT and ARNTL2 were significantly upregulated in LUAD, thus resulted in the poor outcome of LUAD. However, the miR-30b-3p expression was lower in LUAD tissues than that in normal tissues, and high expression miR-30b-3p suggested a better overall survival in LUAD. On the basis of the information discussed before, we believed that there may be a specific link between these three genes, which prompted the subsequent study.

The changes of genetic material itself, including deletion, amplification or mutation, can always be detected in cancer cells, and they seem to directly affect the growth of tumor cells and some basic characteristics of cancer [69]. The DNA methylation is a physiological process programmed during normal development. The epigenetic ground state is formed by step-by-step erasing most methyl groups from the gametic DNA [70]. Although the exact mechanism of this process has not yet elucidated clearly, it is obvious that changes in methylation levels affect cellular physiological processes. Such as, many demethylation processes in tumors have also been observed in normal cells, even in aging Hematopaietic stem cell [71] and epidermal stem cells [72]. Moreover, more and more studies elucidated that methylation are involved in a variety of diseases [73–76], including cancer [76–78]. In our study, the methylation level of ARNTL2 was significantly lower in LUAD tissues than that in normal tissues. This finding suggests that dysregulation of methylation may be associated with the outcome of LUAD. Meanwhile, the correlation analysis between ARNTL2 and methylases suggested that the function of ANRTL2 may be affected by the methylation levels.

It was reported that tumor purity as a confounding factor affects gene expression, which means that the microenvironment changes caused by immune cell infiltration will affect the overall function of cell genome [44]. Then, using TIMER database, we discovered that the copy number of the ARNTL2 was negatively related to the infiltration levels of B cell, CD8 + T cell, macrophage, neutrophil in LUAD. By searching Pubmed, we found that the ARNTL2-related immune process affects several types of diseases, such as LUAD [79, 80], pancreatic cancer [81], type 1 diabetes [82, 83]. These findings imply that ARNTL2-induced alteration may affect the tumor immune microenvironment and the progression of LUAD.

The GSEA and prognosis analysis was performed to investigate the potential mechanisms and functions of hub genes. The results suggested that ARNTL2-related pathways were mainly enriched in cytokine-cytokine receptor interaction, natural killer cell-mediated cytotoxicity, cell cycle, and so on. The enriched immune-related pathways further suggested that immune infiltration play an important role in the progression of LUAD. It is also associated with the prognosis of LUAD.

Besides, our experiment suggested that ITGB1-DT can enhance the proliferation and invasion of H1299 cell via the CCK-8 and transwell assays. Meanwhile, we also found that hsa-miR-30b-3p as a suppressor can inhibit the H1299 cells’ proliferation and invasion. However, several limitations still existed. On the one hand, more experiments need to be performed to confirm the binding between lncRNA, miRNA, and mRNA. And on the other hand, a further study needs to be conducted for the potential mechanisms of the ITGB1-DT/ANRTL2 axis in LUAD.

In summary, our study built a novel ceRNA (ITGB1-DT/miR-30b-3p/ARNTL2) network in LUAD. Additionally, ITGB1-DT/ARNTL2 screened by a series of bioinformatics analyses and experimental validation may be novel biomarkers and prognostic factors in LUAD. And it can be a great reference for future studies, can also help us explore the pathogenesis of LUAD.

## Supplementary Information

**Additional file 1**. Additional figures and tables.

## Data Availability

All data generated or analyzed during this study are included in this article.

## Abbreviations

LUAD: Lung adenocarcinoma
TCGA: The cancer genome atlas
HPA: The human protein atlas
GEO: The gene expression omnibus
DElncRNAs, DEmiRNAs, DEmRNAs: Differentially expressed lncRNAs/miRNAs/mRNAs
GO: Gene ontology
KEGG: Kyoto encyclopedia of genes and genomes
