# Supplementary material for: ITGB1-DT/ARNTL2 axis may be a novel biomarker in lung adenocarcinoma: a bioinformatics analysis and experimental validation
Source: Cancer Cell Int. 2021 Dec 14;21:665. doi: 10.1186/s12935-021-02380-2 (PMC8670189; doi:10.1186/s12935-021-02380-2)
Supplement: Supplementary file 1 — Additional file 1. Additional figures and tables. [file 12935_2021_2380_MOESM1_ESM.pdf]

Supplementary Figure 1

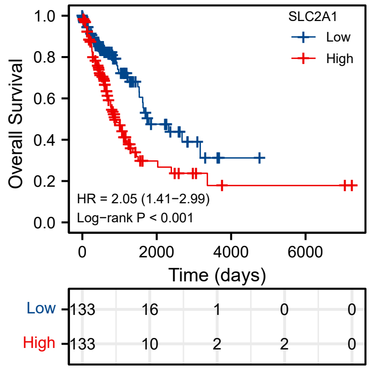

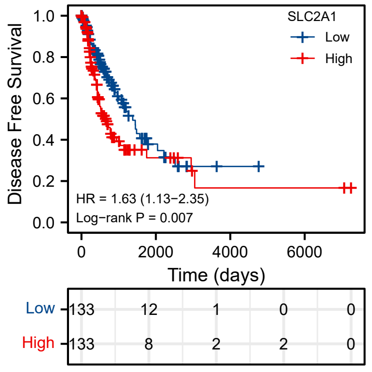

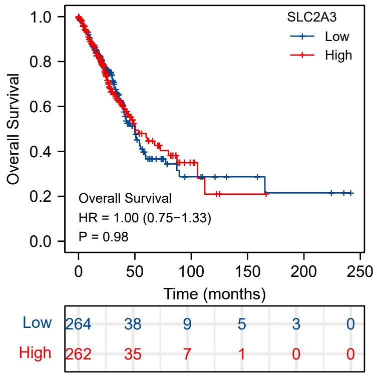

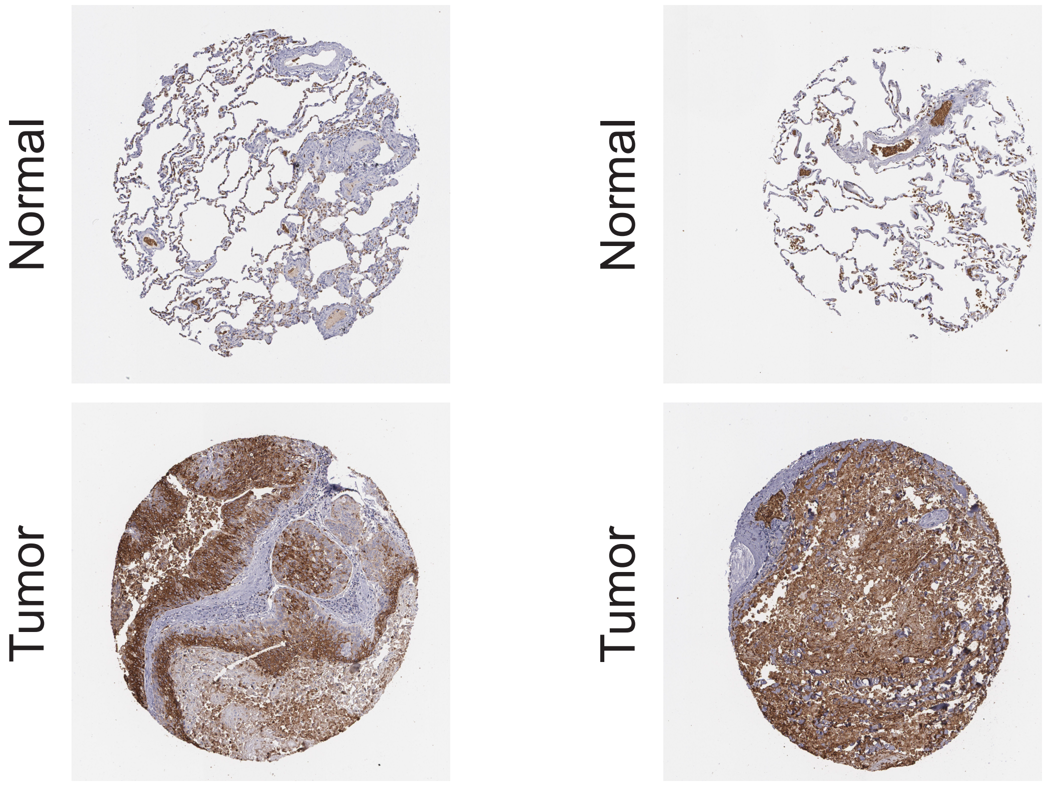

Supplementary Figure 2

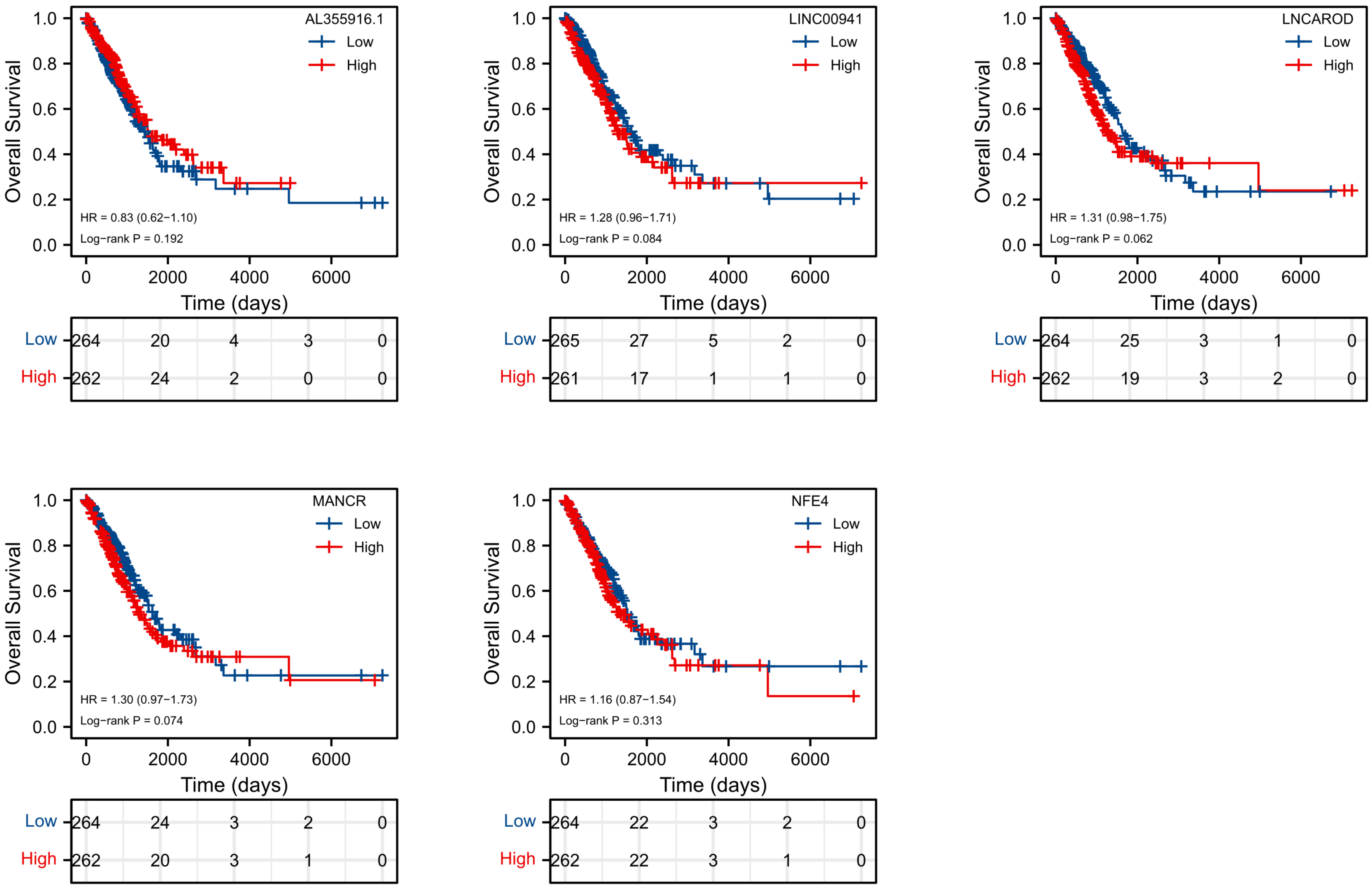

Supplementary Figure 3

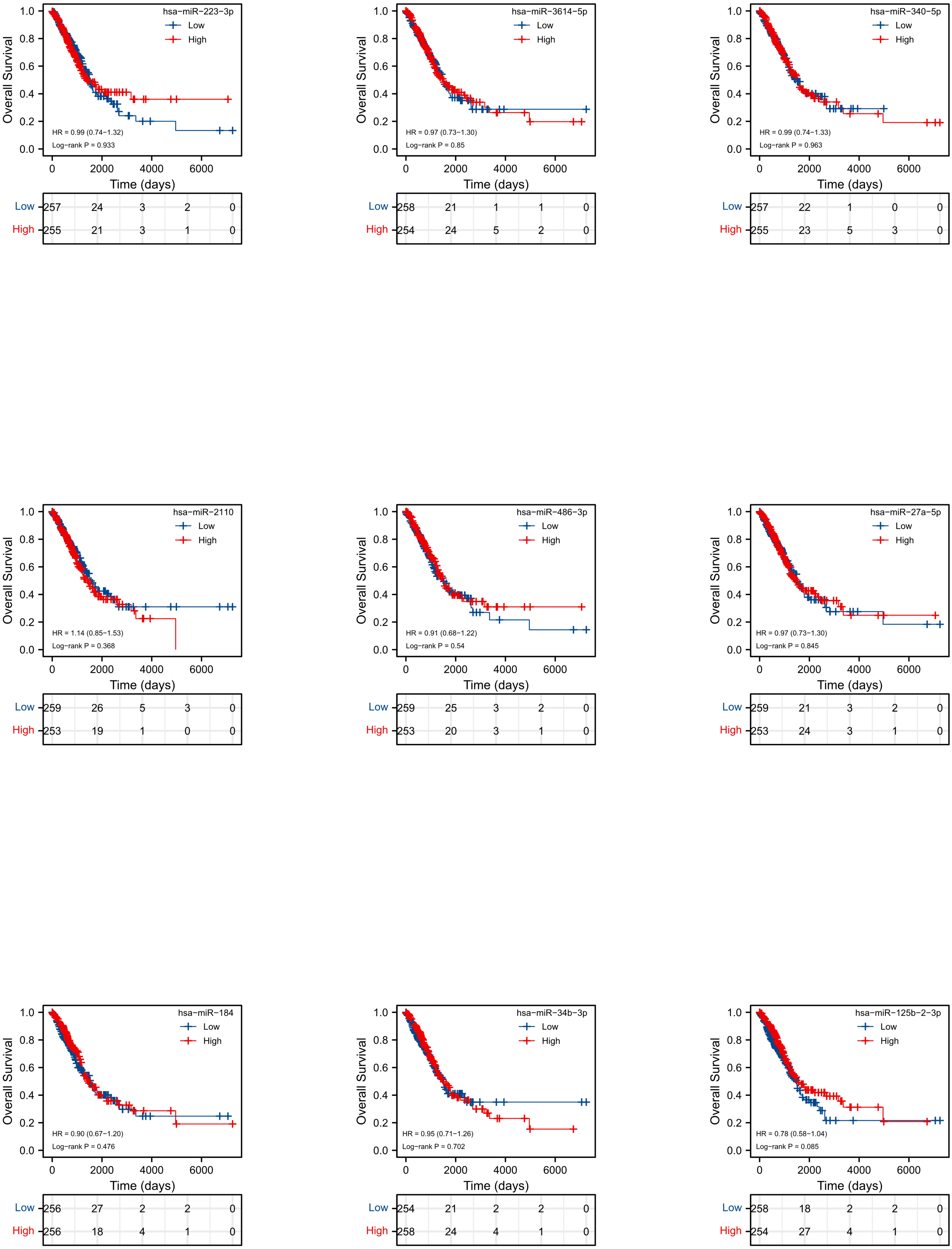

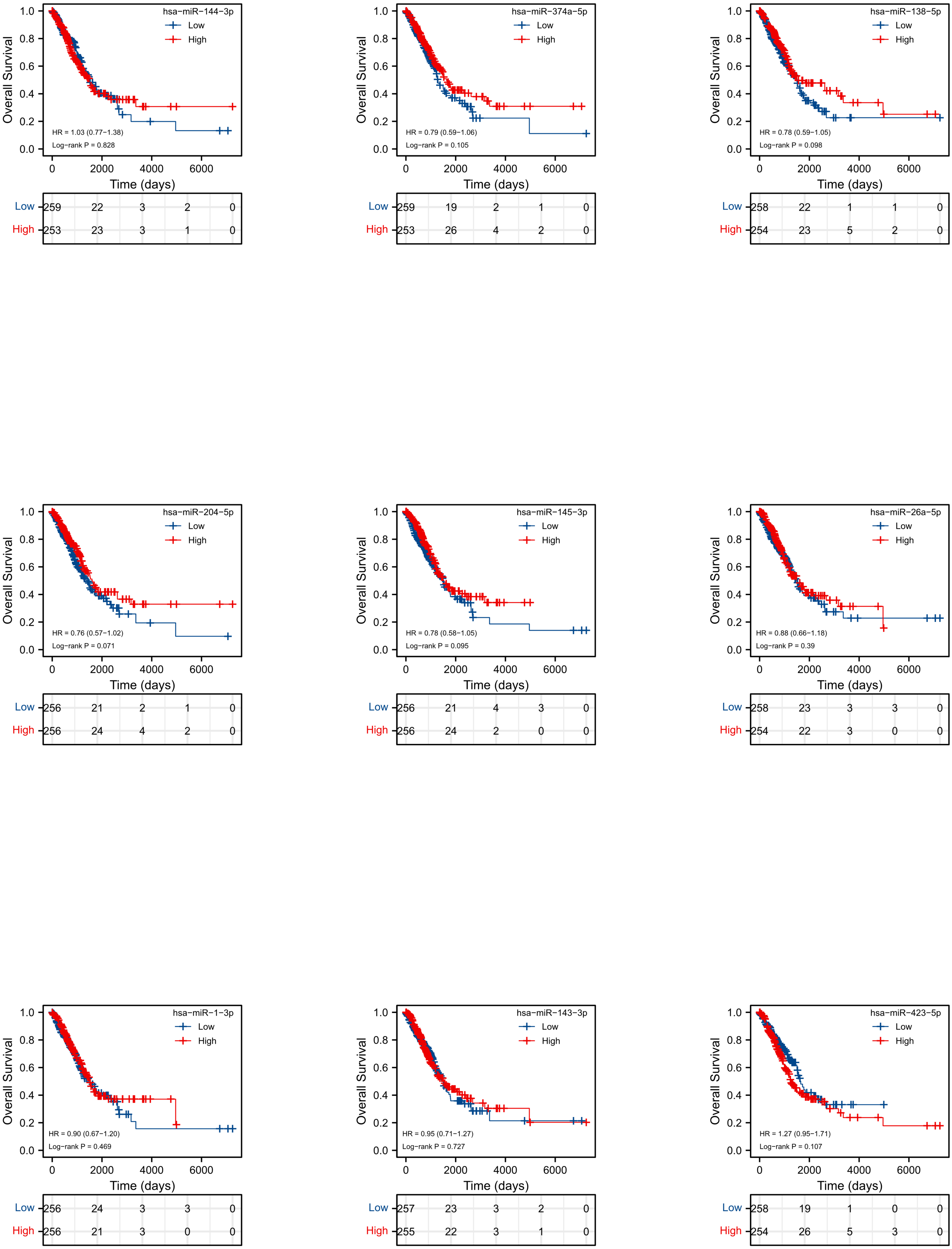
hsa−miR−144−3p
Low
High
HR = 1.03 (0.77−1.38)
Log−rank P = 0.828
Overall Survival
Time (days)
Low 259 22 3 2 0
High 253 23 3 1 0
hsa−miR−374a−5p
Low
High
HR = 0.79 (0.59−1.06)
Log−rank P = 0.105
Overall Survival
Time (days)
Low 259 19 2 1 0
High 253 26 4 2 0
hsa−miR−138−5p
Low
High
HR = 0.78 (0.59−1.05)
Log−rank P = 0.098
Overall Survival
Time (days)
Low 258 22 1 1 0
High 254 23 5 2 0
hsa−miR−204−5p
Low
High
HR = 0.76 (0.57−1.02)
Log−rank P = 0.071
Overall Survival
Time (days)
Low 256 21 2 1 0
High 256 24 4 2 0
hsa−miR−145−3p
Low
High
HR = 0.78 (0.58−1.05)
Log−rank P = 0.095
Overall Survival
Time (days)
Low 256 21 4 3 0
High 256 24 2 0 0
hsa−miR−26a−5p
Low
High
HR = 0.88 (0.66−1.18)
Log−rank P = 0.39
Overall Survival
Time (days)
Low 258 23 3 3 0
High 254 22 3 0 0
hsa−miR−1−3p
Low
High
HR = 0.90 (0.67−1.20)
Log−rank P = 0.469
Overall Survival
Time (days)
Low 256 24 3 3 0
High 256 21 3 0 0
hsa−miR−143−3p
Low
High
HR = 0.95 (0.71−1.27)
Log−rank P = 0.727
Overall Survival
Time (days)
Low 257 23 3 2 0
High 255 22 3 1 0
hsa−miR−423−5p
Low
High
HR = 1.27 (0.95−1.71)
Log−rank P = 0.107
Overall Survival
Time (days)
Low 258 19 1 0 0
High 254 26 5 3 0

Supplementary Figure 4

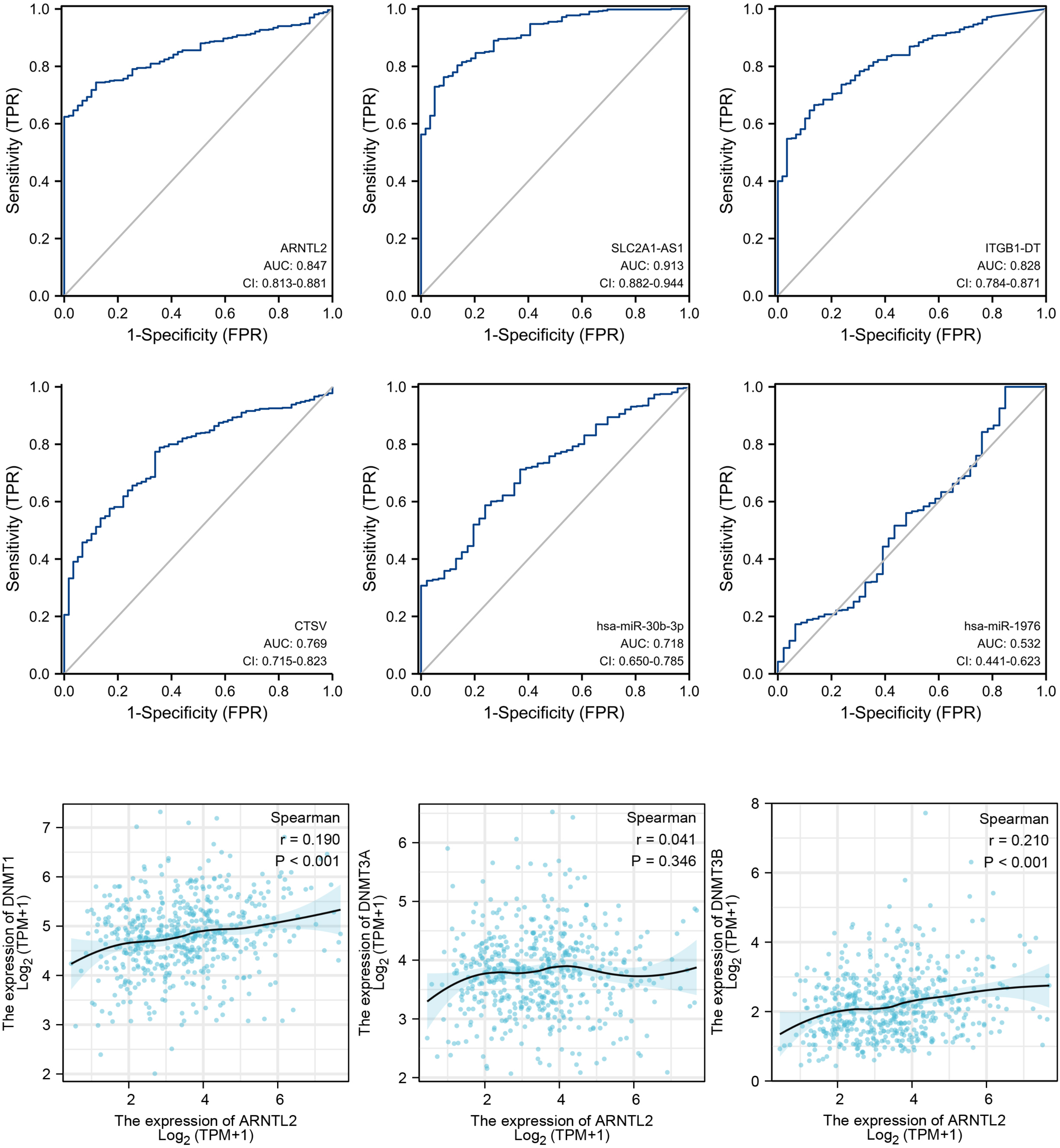

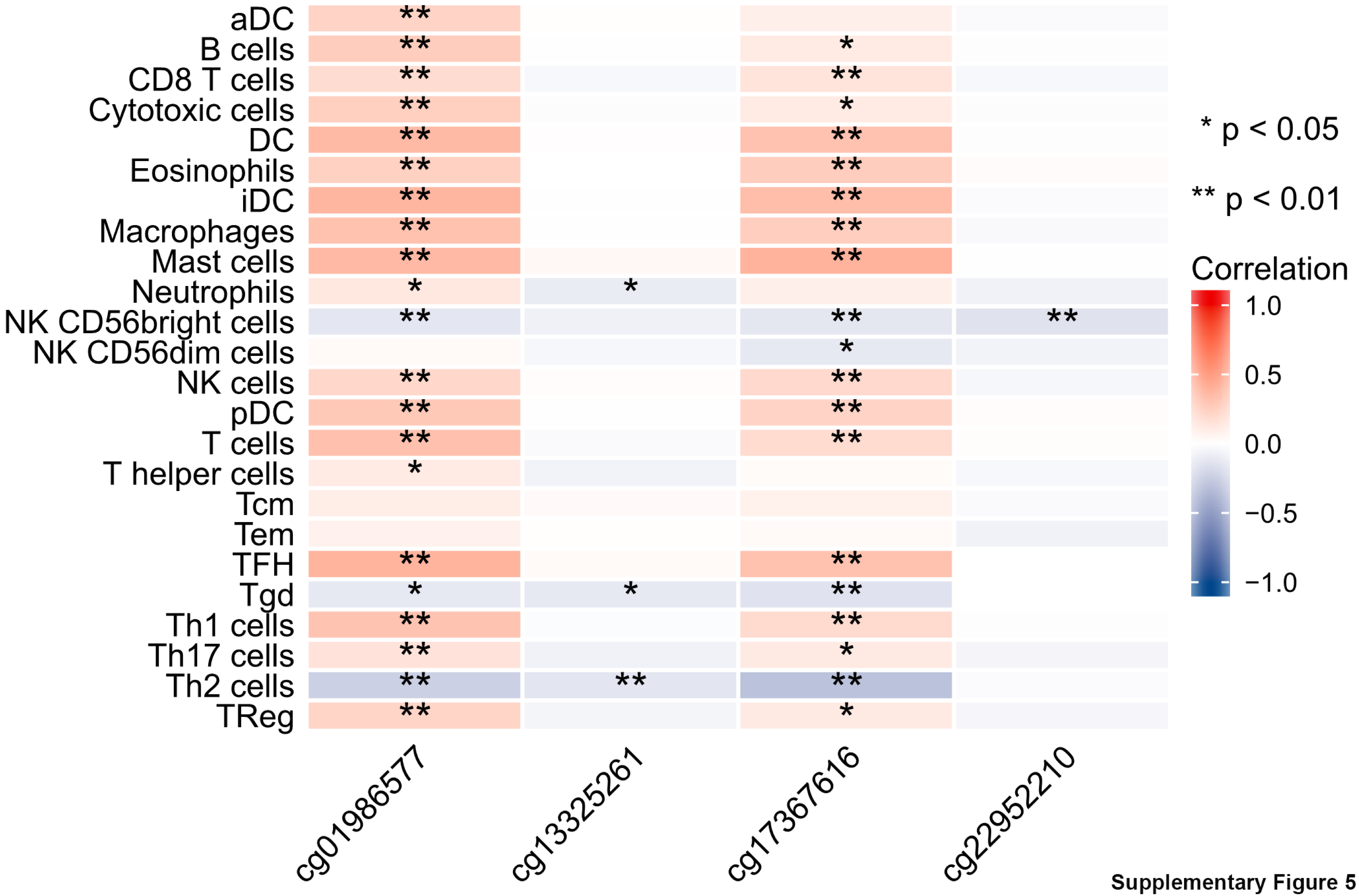

Supplementary Figure 5

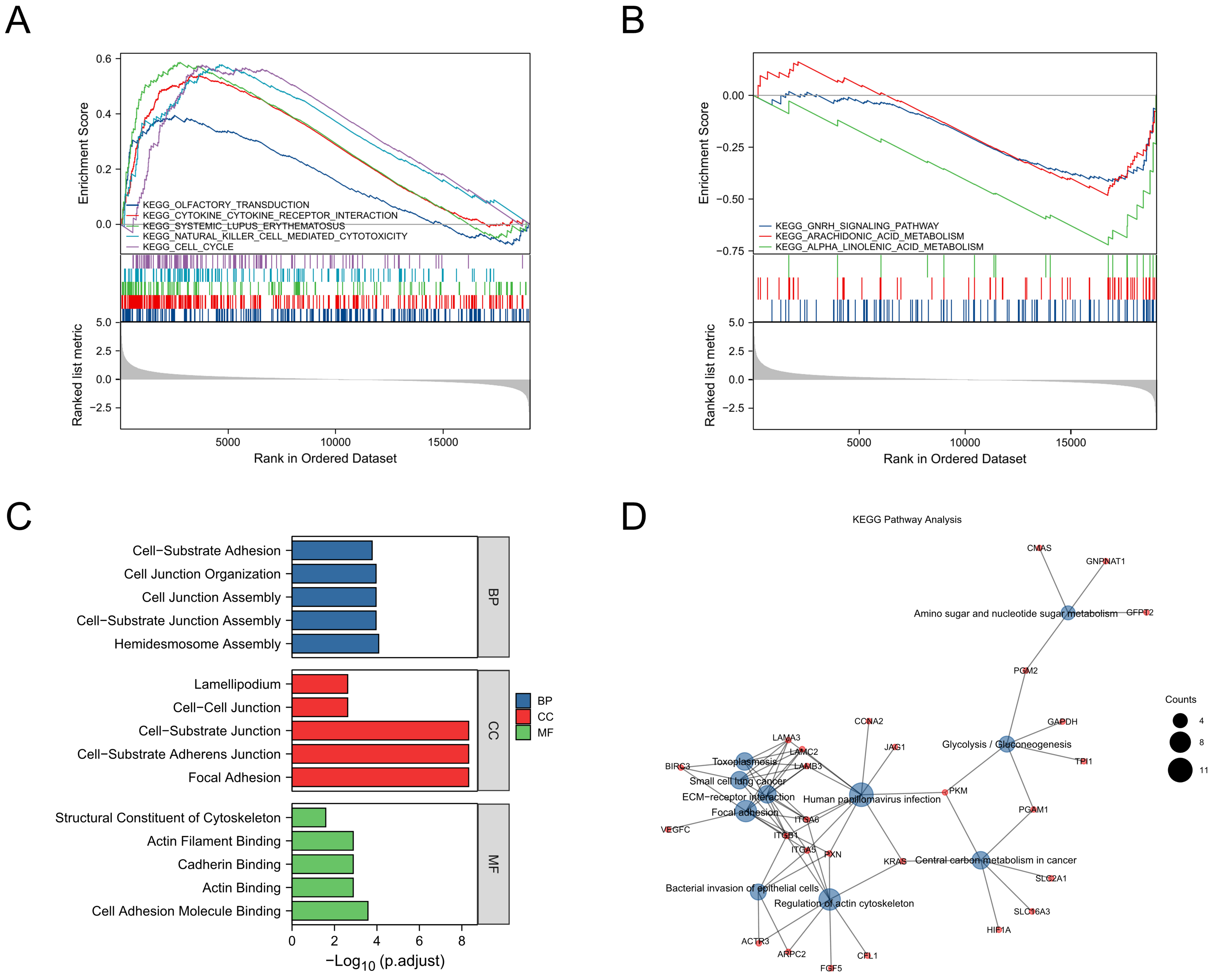

Supplementary Figure 6

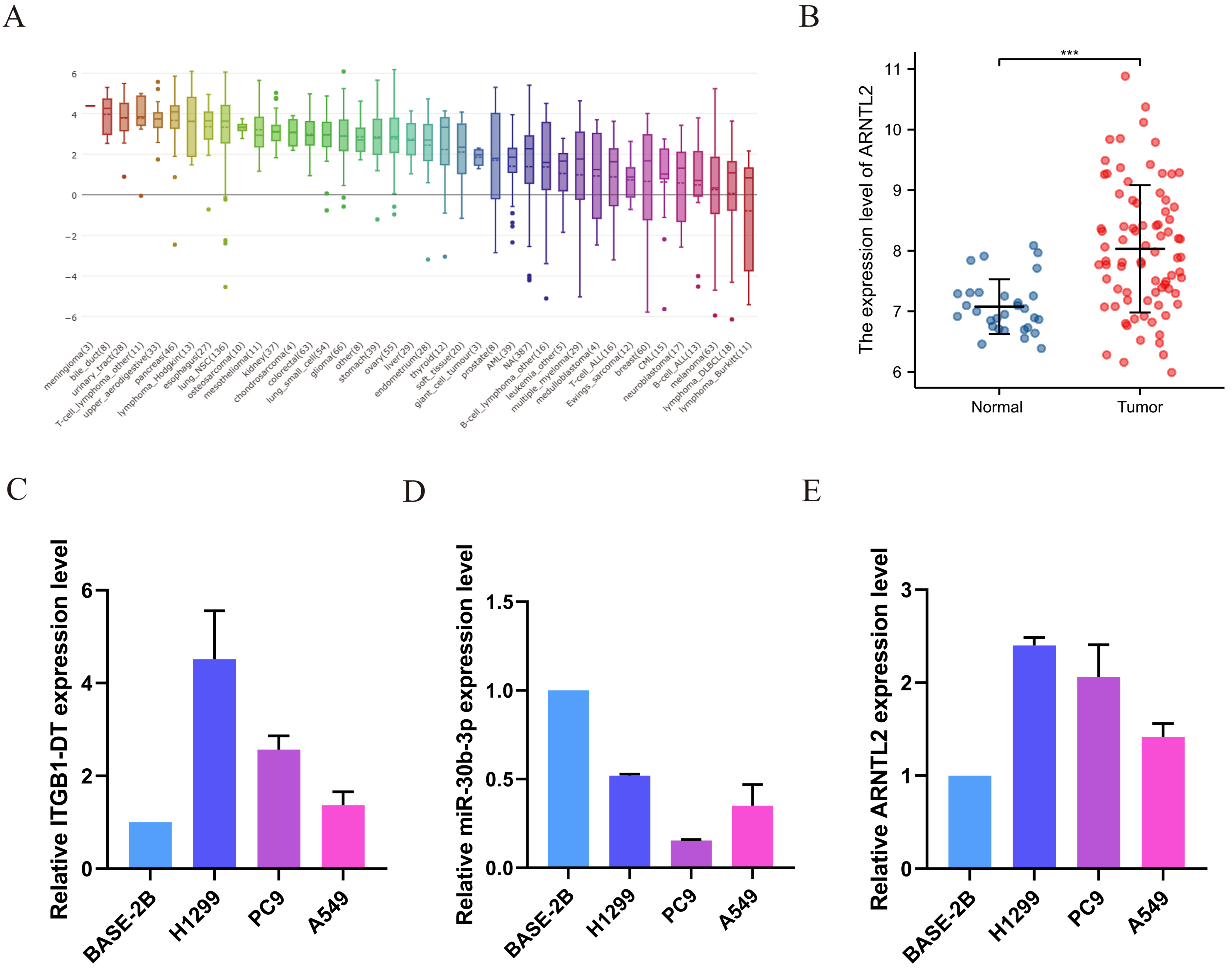

Supplementary Figure 7

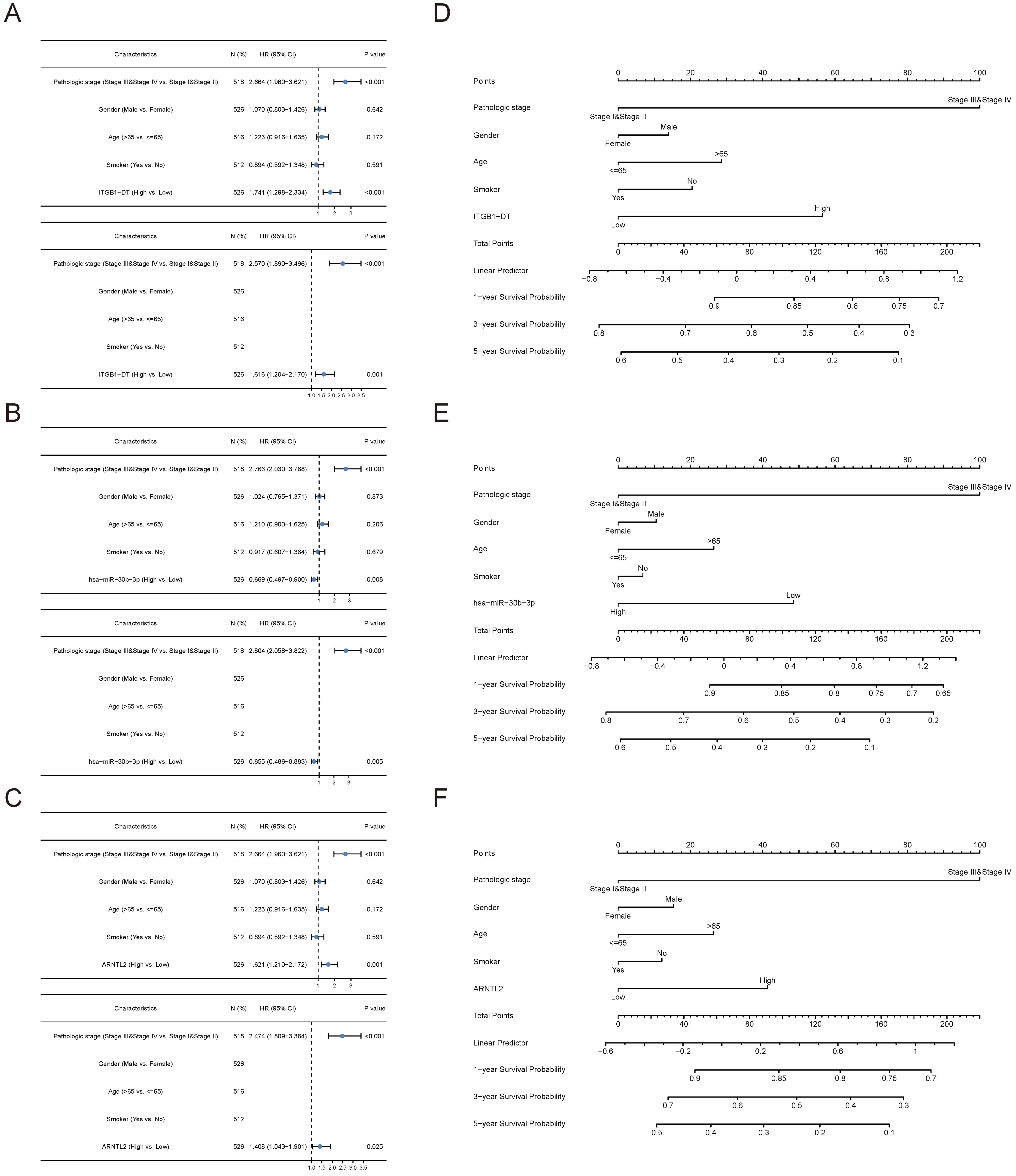

Supplementary Figure 8

Supplementary Figure 9

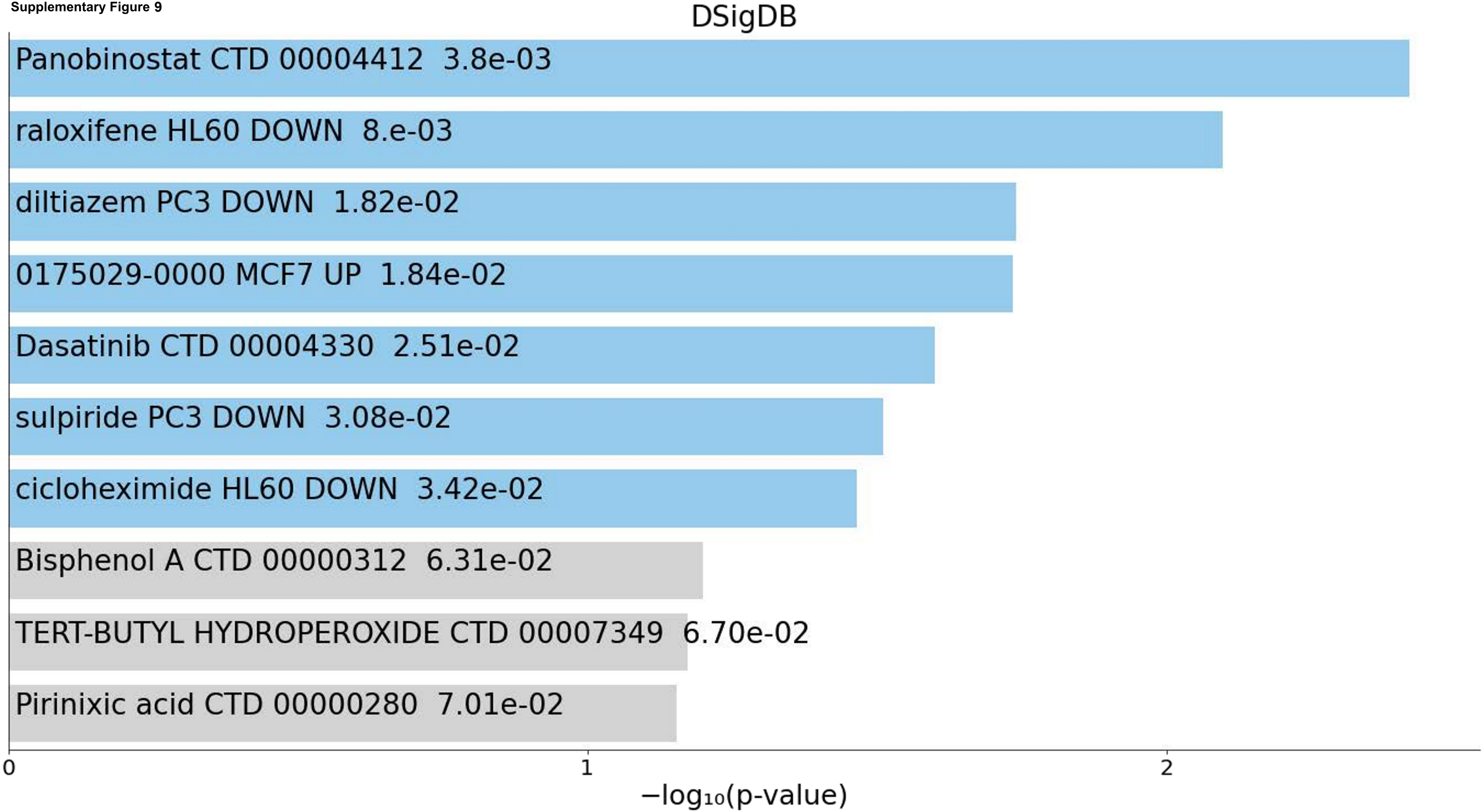

The information of ARNTL2-related drugs.

1. **Panobinostat:** a non-selective histone deacetylase inhibitor used to treat multiple myeloma in combination with other antineoplastic agents.

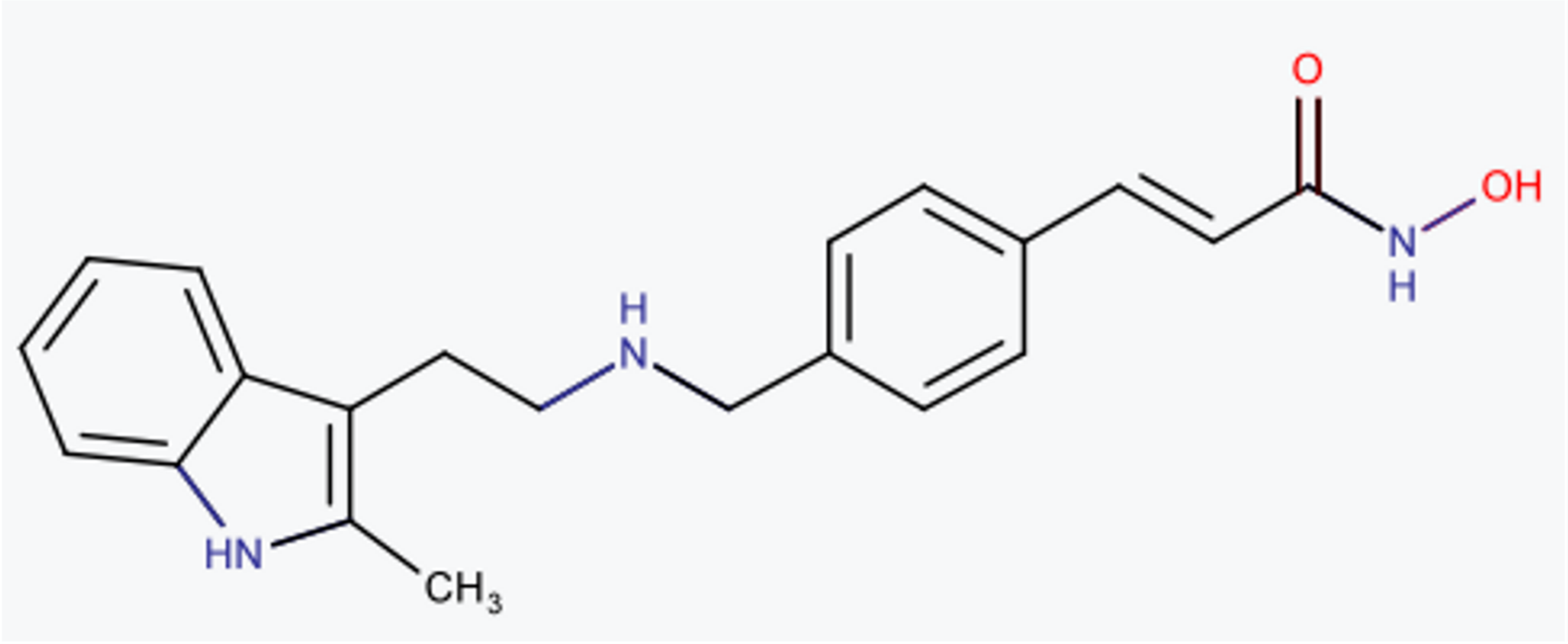

2. **Raloxifene:** a selective estrogen receptor modulator that is used to prevent and treat osteoporosis and reduce the risk of invasive breast cancer in high-risk postmenopausal women.

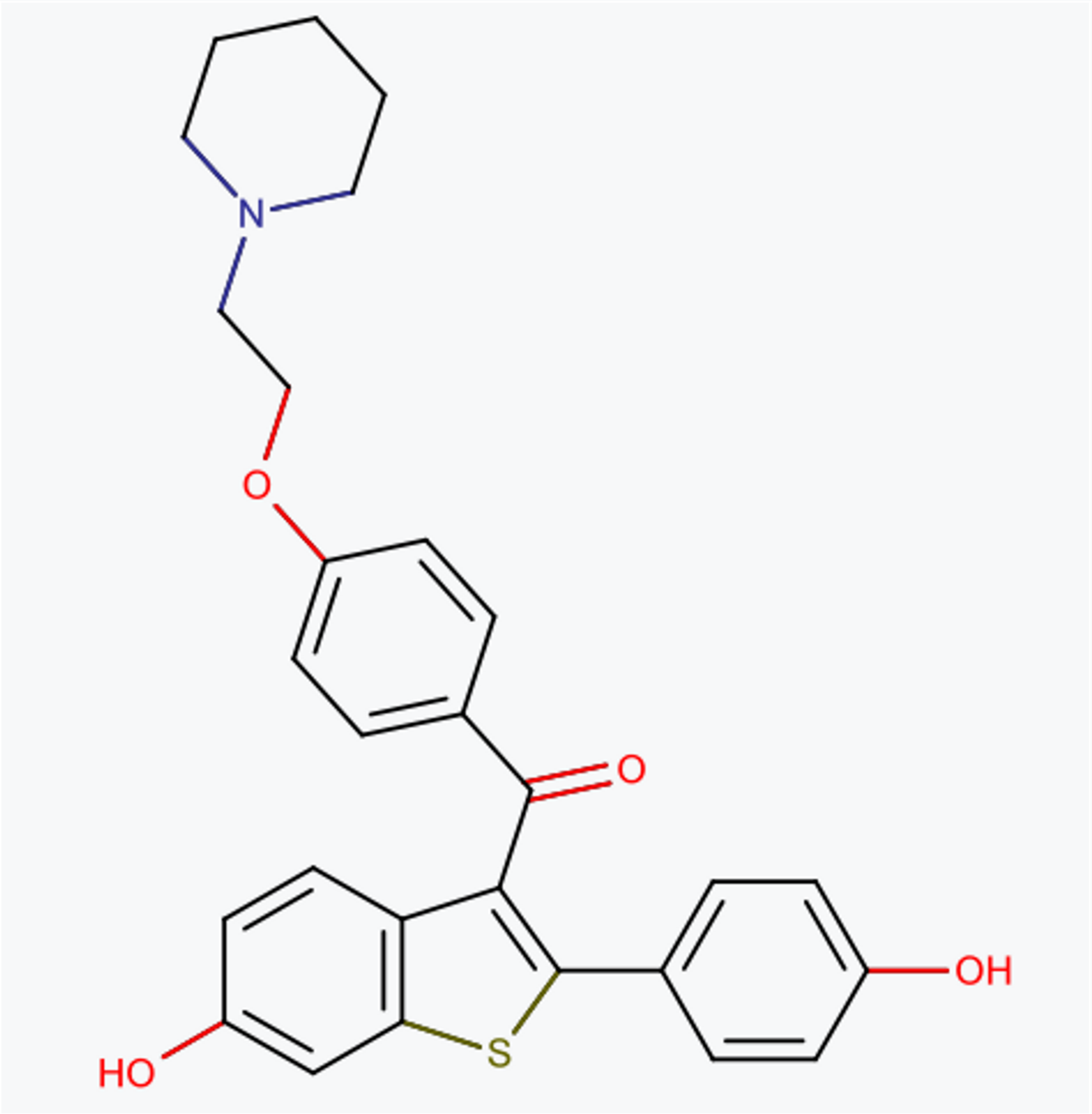

3. **Diltiazem:** a calcium channel blocker used to treat hypertension and to manage chronic stable angina.

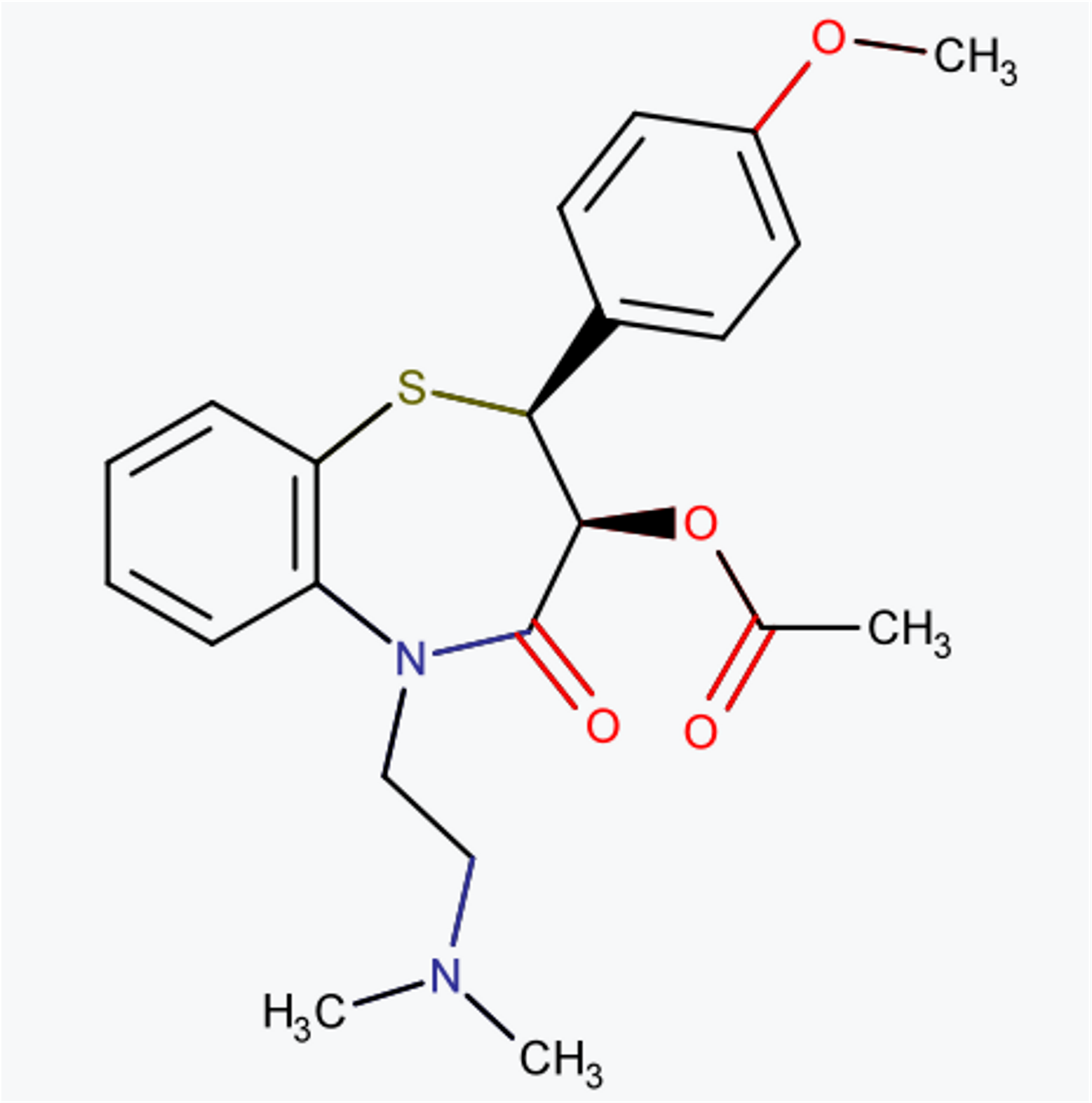

Supplementary Table 1: The sequence of lncRNAs

ENSG00000227533(SLC2A1-AS1):

ATTCGCTGTGTGACTCAGGGCAAGTGTGCGCACCTCTCTGAGCCTCCGCGGCCTTCTCTAG

CAAAATGGTGGAGCCGGTACCCGGCTGTAAGGCAAGCTGGGATGCGCGGAGTAGCGGGTG

GAAAGCTGGACTGGAGTTCTCACTCGGCCGCTGGGTGACTTCGGTGCACTAGGGATAGTAA

CAGTACCACCTCGTAGGGTTGTAGAAAGACCAAGTGAGTTAATATTTGTAAAGTGTTTAGA

ACAGCGTCTACTGCATGGAAAGTAGTATTTATTTGTTAGAGAGACTCCAGGAGGTCTTGCCC

AGGGATTGACTTCTCCCTTCCCTGTCTCCACCCCCCACATTCCCGTTTGTGAGCCAAGCCTT

GCTTTTGCACCGGCCGGGCTCTCTTGCCTGGAAATGCATACCCATCTCAAACCTGGGCTTTA

AAATCACCTCCTCTGGGACGCCTTCCTCTACTTCTCACAGTTCAGGCCTGTTCTGCCCCACA

CAGCAGAAAATGCCCACGATGCAATACCAGAGTCTGCATGGGACCATTTCAACAACCCTTA

GAAGCTGACTCAGCTGTCTCCTGGCCCTGACTTTTCTAAGATGAGAGGAGCTGGTAGGAAC

AATTTGGGAACCCTCAAAGGCTGAGAGTATCCCACCCACTTCACAGATGAAGCAAACTGA

GGCCCAGAGAAGGGAAATTACTTGCCCAAGATCACCCAGCAAGTAAGAAACAGAGCTGGA

GATGAGCTCAGGCCAGCACGGAACCACGCAAACTCCTTCTGTAGGCTTTGTAAAGGATTGA

TGAGAAAAGCAGATGGTTAAGAGCTCTACAACTTCTGTGAGGTAAAGAATATCTGCAGGGC

TGGACGTGGCGGCTCTTGCCTGTAATCTTAGCACTTTGGGAGGCCAAAGTGGGTGGATTGC

TTGAGTCTATGAGTTCAAGACCAGCCTGGGCAACATGGTAAAACTCTGTCTCTCTAAAAAA

AAAAAAAAAAAAAAATTAGCTGAGTGTGGTGTTGTGTGCTTGTAGTCCCAGCTACTTTGGA

GGCTGAAGCGGAAGGATCCCTTGGCCCAGGAGGCAGAGGTTGCAGTGAGCTGAGATTGTG

CCACTGCACTTCAGCTTGGGTGACAGAGCCAGACCTTATTTAAAAAAAAAAAAAGGATATC

TGTGATTACAAGATTACCATGCAATTTTGAAAGCCTGCTGTTGCCCAGCTGGACAGCTAGAG

TTATTCTCAGTGGTGGGATGGCTGAACTGGGGGCCCACTGTGGAGAGCTGGTTTCCCACTG

GAGTTCTCTGAAAAGGTGCCTGATTTCACAGCTCTGATTCTACAGAAGGAGGCCGGACCCT

TCACGTAATTGCTACCCTTTATTTATGGAGCTATTCCTCTGTCGGGATGCCCTTTATATGTGTA

CAGTCTGTGCTGCAGTCGATTCTTATAGTGATTCTATGCAGAAGTTACTTTAGAGGAGGCTG

CTGGGGCGCAGGGTGGGTAGGTACCTTGTCAGCATGGTGGGGCTGGGTCTCCAGCTGGTTG

GGACCCCACCCTCCTATCATGGCCCAGGAGTGAGGCATTCAGGGCTGGACCACCAGCATTT

GTGAAGCTTTTGCCCCATTATCCCATCAGAACATCTTCTTGGCCCTGGCAGGCAGCCAGGGC

AGGGCCATTCTCCACCTTACTGACAAAGAAGCCCAGGGAGGGGAAGAGGGTGCTCCCCCA

ACACCTCCATTGCCTCTGCCTGCTTAGTCCAGAATGCAGGATAGAGAAATAATGGGAAAAG

CTCCCAAGTGTTGCTGGATCTCTGCCTGCCAGGGAAGCTGTGACCCCAGTAAGGGCAGGGT

GTTCCTTCCAGGGAACTCGAGGTGGGGCAGGATGGGTAGGGAGTGAGATCAGAGGGAAGG

CCATGTTGGGAAGATGGGAAGGCAAGTCTCTGGGGGTTTGTGGGATAGAGGAAGGGAACA

TCATAGAACAGGCCAGGAATAGGAGCTTGACCTGGAGATTACTTTGCAGCAGGTTTAAACA

GGGGCCATGTGAGAGTGTGGAGCCCTGGGGTCAGAGGTTACTGGATGCAAATTATGACTCT

GCCACTTATGGGAATGGTGAGTTAGGACAAATGATTTCCCTGGGCCTCAGGGTCTTCATGTA

TAAAAGGGCTGAAGCTCAGTGGGTAACTGGAGCACTGCCTGGGTTAGATCCTGGCTCCACC

CCTTTCCAGTGTGACCTAGGGTGAATGAGTTCACCTCTCTGGGCATCAGTGTCCTTGTCTCT

AAGATGGACTTGATAATAATGAAAGCTACCCCTCACAGGGCTGTGGTGAGGATGAAGGGGT

CTGGCAGCACATGTAGAATTCTTAGAGCAGGCTTTTTTCCTGCAGAGTCTTTGTTGCTATCA

TCATCATCATCATCATCCTCATCCTCATCATCATCTACCTCCAGGGATTCTTGGGGGGCTTAA

ACAAGGCAGGTTGTACAAATTATCTGGCACAGACGTCATTAAATAATAACAGGGCAGAGTG

ACGGGTGCTGTGATACAGGGAAGAGCTGGGAGAGACACAGGAAAGAGAAAAGACTTCTG

TCTGGCTGGGTGGAAGAAGGGTGTGGAAGGTATGGAAGCCAAGGGAAAACTTCCCCTTTG

CCCTCTGAAGGTTCACTGAAAAGTCAACTGACAAAAGGCAGATGAATAGCAGAAATGGCA

TGCAAATTTATTAATGCGCACACAGGGAGAACCACAGAGTGATTGATTACTCCCCACTCTGC

AATGGGGTGCAGAAGCTTATATGCCATCTTGAGGTTACAGAAAGAATGGGGGCTTGGGGCC

TGAGCGCAGTGGCTCATGCCTGTATTCCCACCACTTTGGGAGGCCAAGGTGGGTGGATCAC

CTGAGGTCAGGAGTTTGAGACCACCCTGACCAACATGAAACCCCGTCTCTACTAAAAATAC

AAAAAATTAGCTGGGTGTTGTGGTGCGCACCTGTAATTCCAGCTACTCGGGAGGCTGAGGC

AGGAGAATCACTTGAACCAGGGATGCTGAGGTTGCACGAGCTGAGATCGCACCGCTGCCC

TCCAGCCTGGGCAACAAGAGC

ENSG00000229656(ITGB1-DT):

ACTCTGTAGTCACCCAGGCTGGAATGCAGTGGTGCGATCTTGGCTCACTGCAACCTCCACC

TCCCAGATGCAAGCGATTCTTGTGCCTCAGCCTCCCGAGTAGCTGGGATTAAAGGCATGCG

CCACCACGCCCAGGAGACAGCTTCCCAAGGCTGGAAAGAGGGGAGTGCGGAACATGCCTC

CACCATACCACTGCCCACCTCCTGTGACCTCTGCAGCAAAACCTGAAGCCCCAAAGAGGC

AGCTTCTCAAAGTAAAAGTGGAATGAAGGTCAGAGTCGGGGTTTCACCATGTTGCCCGGCC

TGGTCTCGAACTCCTGGCCTCAAGTCTCTCTGGCTCAGCCTTCCAAAGTGCCAGGATAACA

GGCGTGAACCACGACACCCAGCTTGCTTTGTGTACTTCTAGGGGCTCAGGTGAACCACATG

AGTTCCTGGATGTGCTTCCATGTTTCACAGTGAATTCATGTTTTTGGTCCAATCATGACCATT

TAGACTGTTTAAGAAGGCTGTGTACCAGCTTTAATATTGTATTTTATGATATTAGTGATCCCTG

AATTTTATTTGAAAAAATATTATTTTACTGAGTGATTTAGTAGGCTTGATCATTTTCCTTGCAG

CTTTCATATTTTGTGACACACTGATTTAAGAATCAAGTCTATATCTGAATGTGAAAAATCTGC

AATGAGGATAGCAAACAATTATATTGTTTTAATAGAAAAATTATTTGATAATTTCCATGTATTG

ATGATTTCAGTAGAATCTATTCTGCATTTAACCAACAATCAGTAGTTCCCAAGCTATTCCGTA

GTTTTAGCTTTAACACATAGTACATGCTTAATAAACATTAGCTGAATGAATGAATGAATGAAT

GACTAAAGACTTTCAGGACCTGGAAGGGAGGATGTAAAGAACCCAGGGCCAAGTG

Supplementary Table 2: The sequence and methylation of promoter

cg01986577: R = -0.331 P < 0.001

Sequence around CpG island:

TAGGGCTGTCAAGTGATGCCCAAAAGATCTTGTTTCTGGATTTACCCAACC
GTTCCAGGAGGCAAAACGGCTTCCTCGGGACCCTCTCTCTGTCATCTACA

cg17367616: R = -0.357 P < 0.001

Sequence around CpG island:

GACGAGAGACCCTGCTCTTTTCTGCTTAAGGAAGGGCTTAGCTGCAGAGA
CGAGAGTGTAGATGACAGAGAGAGGGTCCCGAGGAAGCCGTTTTGCCTCC
T

cg22952210: R = -0.167, P < 0.001

Sequence around CpG island:

TTTTAGTGCTTTGCTGCTCGCGGGAGGGCTCGGCGGGGCGGTGTCCGCAGC
GACTCCGCCCTTGGCCCGGGTCTCTTTCCCGCCGGGAAGCCCCGCGGCGC

cg13325261: R = -0.149, P < 0.01

Sequence around CpG island:

CTGCGGCGGGAGGTGAGGTTGCCGGTGGCGAGGCGACGGCCCCAGGTGG
GCGCGCGGCGTCTGACGCGCTGGGGGCGTCCCCGCGACGCCAGGTCTGCC
CC

Term	P value	Combined Score	Genes
**Panobinostat CTD 00004412**	0.0038	111031.8	ARNTL2
**raloxifene HL60 DOWN**	0.008	95793.97	ARNTL2
**diltiazem PC3 DOWN**	0.0182	78668.52	ARNTL2
**0175029-0000 MCF7 UP**	0.0184	78437.93	ARNTL2
**Dasatinib CTD 00004330**	0.0251	71848.06	ARNTL2
**sulpiride PC3 DOWN**	0.03085	67426.17	ARNTL2
**cicloheximide HL60 DOWN**	0.0342	65201.84	ARNTL2
**Bisphenol A CTD 00000312**	0.0631	51773.81	ARNTL2
**TERT-BUTYL HYDROPEROXIDE CTD 00007349**	0.06705	50422.59	ARNTL2
**Pirinixic acid CTD 00000280**	0.0701	49430.43	ARNTL2

Supplementary Table 3: The ARNTL2-related drugs

Supplementary Table 4: The primers of lncRNA, miRNA, and mRNA

Name	Primer
**ITGB1-DT**	F: 5’-CCTGTACGTGACTGGTGGAG-3’<br>R:5’-TCTGTCCTTGCAACAGTGCT-3’
**hsa-miR-30b-3p**	F: 5’-AACAATCTGGGAGGTGGATGTTTAC-3’
**ARNTL2**	F: 5’-AGAGCATGGATGCTTACCCA-3’<br>R: 5’-TTGGAGGCCAGCTTCTCAAG-3’

Supplementary Figure Legend

Supplementary Figure 1: **The survival analysis of SLC2A1 and SLC2A3 (GLUT3) in LUAD patients.**

Supplementary Figure 2: The survival analysis of DElncRNAs in LUAD.

Supplementary Figure 3: The survival analysis of DEmiRNAs in LUAD.

Supplementary Figure 4: The ROC curve of 6 hub genes and correlation between ARNTL2 and methyltransferase.

Supplementary Figure 5: The correlation between promoter methylation level of ARNTL2 and immune infiltration.

Supplementary Figure 6: Functional enrichment analysis of ARNTL2. (A and B) The GSEA analysis of ARNTL2. **(C and D)** The KEGG and GO enrichment analysis of 200 ARNTL2-related genes.

Supplementary Figure 7: Validation of ARNTL2 expression in LUAD. (A) Expression of ARNTL2 in pan-cancer cell lines, and ARNTL2 is frequently highly expressed in lung cancer cell lines. **(B)** The validation of abnormal ARNTL2 expression in GSE43458 LUAD microarray. **(C-E)** The validation of abnormal ITGB1-DT, has-miR-30b-3p, and ARNTL2 expression in BEAS-2b, H1299, PC9, and A549 cell lines.

Supplementary Figure 8: Prognostic analysis in LUAD. (A-C) Univariate and multivariate Cox regression analysis. **(D-F)** Prediction model of prognostic factors.

Supplementary Figure 9: Prediction of drugs.
